# A chemiluminescent probe for cellular peroxynitrite using a self-immolative oxidative decarbonylation reaction†
†Electronic supplementary information (ESI) available: Additional experimental details, supplementary figures, and scanned spectra. See DOI: 10.1039/c7sc05087a


**DOI:** 10.1039/c7sc05087a

**Published:** 2018-01-31

**Authors:** Jian Cao, Weiwei An, Audrey G. Reeves, Alexander R. Lippert

**Affiliations:** a Department of Chemistry , Southern Methodist University , Dallas , TX 75275-0314 , USA . Email: alippert@smu.edu; b Center for Drug Discovery , Design, and Delivery (CD4) , Southern Methodist University , Dallas , TX 75275-0314 , USA; c Center for Global Health Impact (CGHI) , Southern Methodist University , Dallas , TX 75275-0314 , USA

## Abstract

Peroxynitrite (ONOO^–^) is a highly reactive oxygen species which has been recognized as an endogenous mediator of physiological activities like the immune response as well as a damaging agent of oxidative stress under pathological conditions. While its biological importance is becoming clearer, many of the details of its production and mechanism of action remain elusive due to the lack of available selective and sensitive detection methods. Herein, we report the development, characterization, and biological applications of a reaction-based chemiluminescent probe for ONOO^–^ detection, termed as **PNCL**. **PNCL** reacts with ONOO^–^*via* an isatin moiety through an oxidative decarbonylation reaction to initiate light emission that can be observed instantly with high selectivity against other reactive sulphur, oxygen, and nitrogen species. Detailed studies were performed to study the reaction between isatin and ONOO^–^, which confirm selectivity for ONOO^–^ over NO_2_˙. **PNCL** has been applied for ONOO^–^ detection in aqueous solution and live cells. Moreover, **PNCL** can be employed to detect cellular ONOO^–^ generated in macrophages stimulated to mount an immune response with lipopolysaccharide (LPS). The sensitivity granted by chemiluminescent detection together with the specificity of the oxidative decarbonylation reaction provides a useful tool to explore ONOO^–^ chemistry and biology.

## Abstract 2

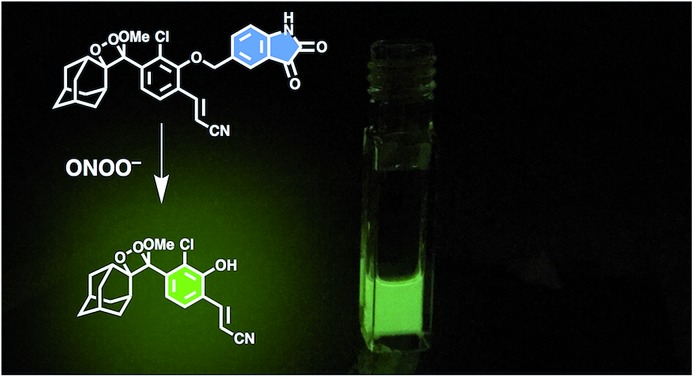
Peroxynitrite is a damaging agent of oxidative stress that has been difficult to monitor in living cells. Here, an isatin-based chemiluminescent probe for peroxynitrite is reported.

## Introduction

Peroxynitrite (ONOO^–^) is a highly reactive oxygen species that can be formed biologically from a diffusion-controlled reaction between superoxide (O_2_˙^–^) and nitric oxide (NO˙).^1^ In biological systems, ONOO^–^ has long been known as a deleterious species due to its oxidative damage to lipids, proteins, and nucleic acids.^2^ Abnormal regulation of ONOO^–^ in living systems is associated with diseases such as ischemia-reperfusion,^3^ diabetes,^4^ cardiac dysfunction,^5^ inflammatory conditions,^6^ autoimmune, and neurodegenerative diseases.^7^,^8^ However, ONOO^–^ has been proposed to act as a physiological mediator in certain contexts, for example in immune system function^9^ and ischemic preconditioning.^10^ Macrophages produce nitric oxide and superoxide to form ONOO^–^ to attack invading pathogens.^11^ Macrophage cells recognize lipopolysaccharide (LPS), a component of the bacterial cell wall, which induces high expression of inducible nitric oxide synthase (iNOS).^12^ The effect of LPS is amplified by release of IFN-β, providing a mechanism for robust local production of NO˙ and ONOO^–^. Although important biological functions of ONOO^–^ are still emerging, there remains a lack of efficient and selective methods to monitor its production in real time to study its effects in living systems. Therefore, the development of precise approaches for detecting ONOO^–^ is crucial to provide a more detailed understanding of its complex biological effects.

Much of the difficulty of studying the biological roles of ONOO^–^ stems from its complex chemistry. ONOO^–^ coexists with its protonated form ONOOH (p*K*_a_ 6.8) under physiological pH, and can directly undergo two-electron reactions with thiols,^13^ CO_2_,^14^ and carbonyls.^15^ The H^+^ and CO_2_ adducts, ONOOH and ONOOCO_2_^–^, rapidly decompose *via* homolytic cleavage reactions to form secondary radicals such as hydroxyl radical (HO˙), carbonate radical (CO_3_˙^–^), and nitrogen dioxide (NO_2_˙), which mediate potent one-electron chemistry, including tyrosine nitration.^16^ ONOO^–^ can also form from reaction between O_2_ and NO^–^ produced by photolysis of Angeli's salt under alkaline conditions.^17^ Under physiological conditions, Angeli's salt has been widely used as a nitroxyl (HNO) donor for biological studies. Reaction of HNO with O_2_ also proceeds at neutral pH to form a potent oxidative species and represents an important decomposition pathway of HNO.^18^,^19^ While there are similarities and differences between ONOO^–^ and the autooxidation product of HNO formed at neutral pH, the details of this chemistry and the exact species eliciting the oxidative effect of Angeli's salt is still under debate.

The classical approach for studying biological ONOO^–^ relies on immunostaining of 3-nitrotyrosine residues,^20^ which lacks temporal precision and is an indirect method tracking the “footprint” of ONOO^–^. A large number of reaction-based fluorescent probes have been developed in an attempt to solve this problem. These small molecule tools can react with chemical species as they are produced, enabling detection within living intact systems. Several families of reaction-based fluorescent probes for ONOO^–^ detection have been reported and applied in biological studies, such as trifluorocarbonyl-based,^21^*N*-dearylation-based,^22^ and boronic ester-based^23^ probes. Organoselenium-based,^24^ and organotellurium-based^25^ probes are advantageous because the fluorescence signal is reversible, but depend upon reaction with cellular reductants to reverse the signal.^26^ This limited reversibility, as well as other intrinsic drawbacks of fluorescent detection methods such as photobleaching and autofluorescence highlights the need for new methods of ONOO^–^ detection. Chemiluminescence provides an attractive solution for studying biological molecules and processes due to the low background, high signal-to-noise ratio, and reversible signal of this technique.^27^ Nanoparticle-based methods have recently been reported for chemiluminescent ONOO^–^ detection.^28^,^29^ These probes rely on an interesting radical-pair mechanism to generate signal from the ONOO^–^ decomposition products HO˙ and O_2_˙^–^. Although these systems do display good sensitivity, complementary approaches that operate *via* direct reaction with ONOO^–^ are desirable. Chemiluminescent probes based on sterically hindered 1,2-dioxetanes offer opportunities to incorporate specific reaction-based triggers and have already been applied for detection of analytes in cellular systems and for live animal imaging.^30^–^34^ One fruitful design strategy uses Schaap's adamantylidene-dioxetane attached to an analyte-specific reactive handle.^35^ A striking substituent effect for Schaap's 1,2-dioxetanes has been reported, where incorporation of an electron withdrawing group such as an acrylonitrile at the *ortho* position of the phenol results in a significant increase in the overall chemiluminescent quantum yield of 1,2-dioxetanes.^36^ As a result, chemiluminescent emission can be observed in aqueous conditions without the addition of any polymeric enhancer solutions. We herein describe the development and application of a selective chemiluminescent probe for ONOO^–^ detection, **PNCL**, with both *in vitro* and live cell applications.

## Results and discussion

### Design and synthesis of **PNCL**

**Scheme 1:**
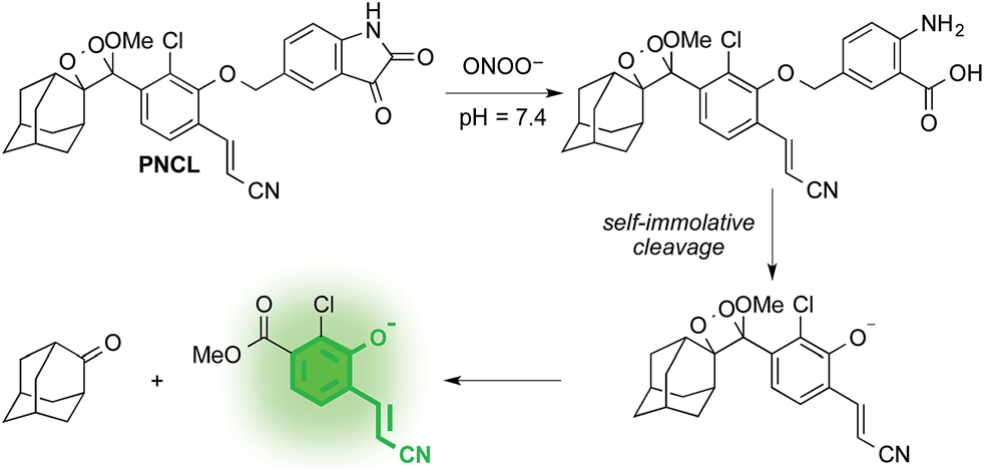
Spiroadamantane 1,2-dioxetane-based probe for chemiluminescent ONOO^–^ detection.

**Scheme 2:**
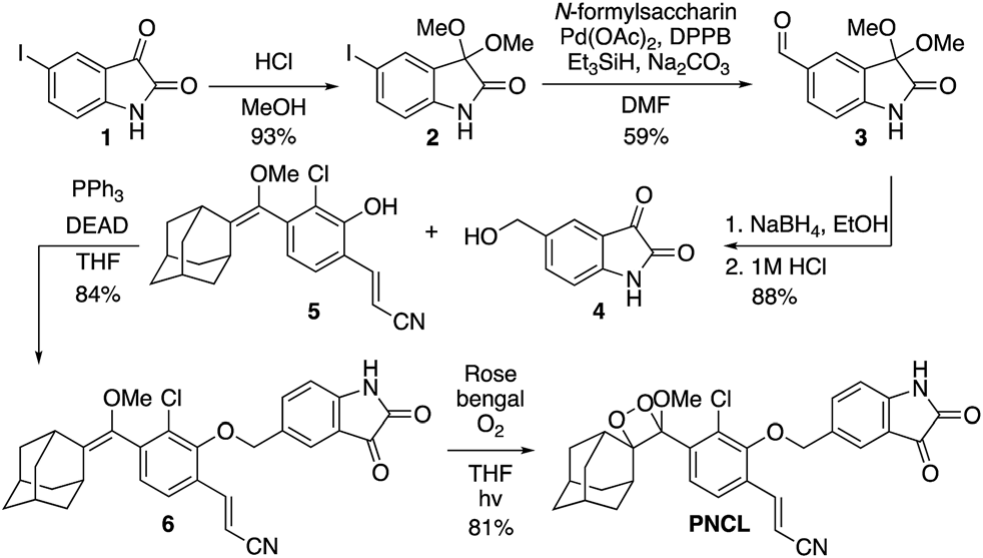
Synthesis of **PNCL**.

As part of a program to develop chemical probes for imaging reactive sulphur,^29^,^37^ oxygen,^30^,^38^,^39^ and nitrogen species, our laboratory has previously reported that isatin reacts with ONOO^–^*via* an oxidative decarbonylation reaction to generate anthranilic acid.^40^^19^F NMR probes, 5-fluoroisatin and 6-fluorisatin, have been developed for selective detection of ONOO^–^ that utilize this newly discovered transformation. In order to expand this reaction-based detection strategy for luminogenic detection, we adopted a self-immolative 1,6-elimination strategy for chemiluminescent ONOO^–^ detection that relies on tethering an isatin moiety to a 1,2-dioxetane chemiluminescent scaffold *via* an ether linkage (Scheme 1). The isatin serves as the reaction handle, and 1,6-elimination can be triggered after reacting with ONOO^–^ to form the anthranilic acid with the amino group positioned *para* to the benzylic ether. Luminescent emission is then initiated upon the decomposition of the 1,2-dioxetane through a chemically initiated electron exchange luminescence (CIEEL) mechanism. Synthesis of a peroxynitrite chemiluminescent probe (**PNCL**) proceeded from commercially available 5-iodoisatin **1** by protection of the ketone group as the dimethylacetal **2** (Scheme 2). The protected 5-iodoisatin was subjected to palladium catalyzed reductive formylation with *N*-formyl saccharin as the CO source to form the aldehyde **3**,^41^ followed by reduction of the aldehyde with NaBH_4_ and deprotection to afford benzyl alcohol **4**. The alcohol **4** was linked to the phenol **5** (ref. 36) through a Mitsunobu reaction to deliver the enol ether precursor **6**. This precursor underwent [2 + 2] cycloaddition with photogenerated singlet oxygen using Rose bengal as a sensitizer to provide the final 1,2-dioxetane **PNCL**.

### Spectral response of the probes to ONOO^–^

**Fig. 1:**
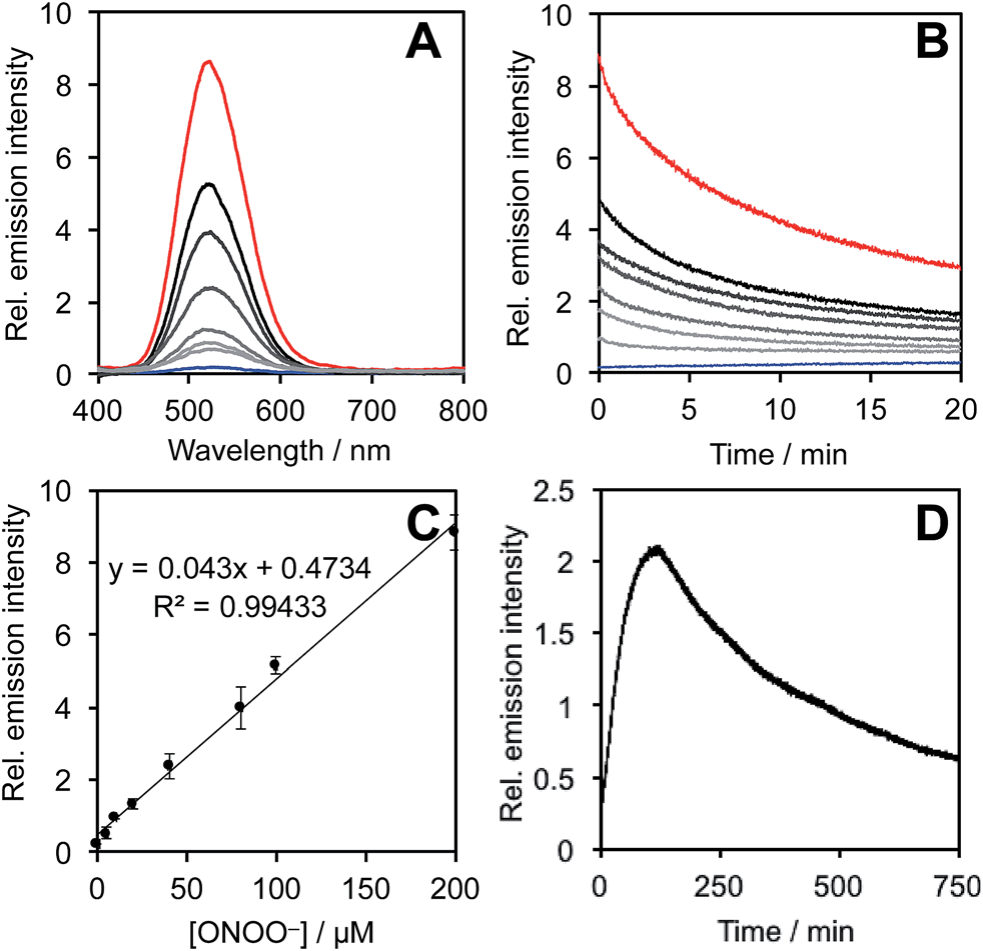
Response of **PNCL** to ONOO^–^. (A) Chemiluminescent emission spectra of 20 μM **PNCL** and 0 (blue trace), 5, 10, 20, 40, 80, 100, 200 μM (red trace) ONOO^–^. (B) Time scans of the chemiluminescent emission at 525 nm of 20 μM **PNCL** and 0 (blue trace), 5, 10, 20, 40, 80, 100, 200 μM (red trace) ONOO^–^. (C) Peak emission intensity at 525 nm of 20 μM **PNCL** after adding 0–200 μM ONOO^–^. (D) Time scan of the chemiluminescent emission at 525 nm of 20 μM **PNCL** and 200 μM SIN-1. All experiments were performed in 20 mM HEPES (pH 7.4), containing <1% DMSO.

After obtaining **PNCL**, its photophysical properties and reactivity towards ONOO^–^ were characterized. Luminescence emission spectra were collected using an F-7000 Hitachi spectrophotometer by treating 20 μM **PNCL** with 0–200 μM ONOO^–^ (Fig. 1). An emission peak centered at 525 nm was observed in the presence of ONOO^–^ with ∼28-fold increase in peak luminescence intensity compared to a blank control (Fig. 1A). The reaction between **PNCL** and ONOO^–^ was analyzed by GC-MS, confirming the generation of 2-adamantanone and 2-chloro-4-acrylonitrile-3-(methoxycarbonyl)phenol (Fig. S1†). The luminescence emission slowly decayed over a course of 20 min (Fig. 1B), consistent with fast reaction kinetics of ONOO^–^ with the isatin moiety of **PNCL**,^40^ followed by a slower 1,6-elimination. Interestingly, ONOO^–^ can be added to a solution of **PNCL** over several cycles with good recovery of chemiluminescent signal (Fig. S2†). This shows that the reversible chemiluminescent emission has good reproducibility and can be used monitor ONOO^–^ fluxes over time. The dose dependence of the peak chemiluminescent emission at 525 nm measured 10 seconds after ONOO^–^ addition showed excellent linearity, demonstrating that ONOO^–^ can be quantified using **PNCL** (Fig. 1C). Calibration of the response of **PNCL** with lower concentrations of ONOO^–^ revealed a detection limit of 6 nM (3*σ*) bolus ONOO^–^ (Fig. S3†), comparing favorably to previous fluorescent and chemiluminescent methods (Table S1†). The reactivity of **PNCL** towards SIN-1 was also evaluated (Fig. 1D), showing that **PNCL** can monitor the slow release of ONOO^–^ over time, as would be observed in biological systems.

**Fig. 2:**
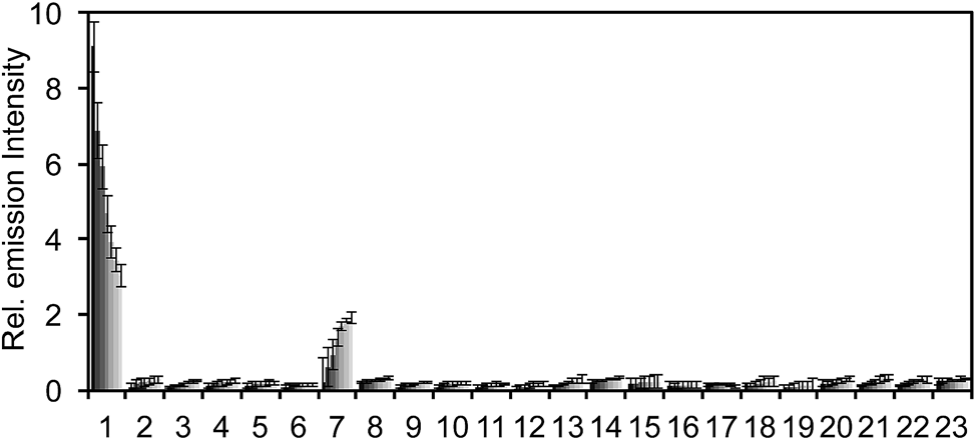
Selectivity of **PNCL***versus* cations and reactive sulphur, oxygen, and nitrogen species. Chemiluminescent emission at 525 nm of 20 μM **PNCL** and 200 μM biologically relevant analytes in 20 mM HEPES (pH 7.4). Bars represent chemiluminescent emission at 525 nm at 4, 8, 12, 16, 20 min after addition of reactive species. Legend: (1) ONOO^–^, (2) Cys, (3) DEA NONOate, (4) GSH (5 mM), (5) GSNO, (6) H_2_O_2_, (7) Angeli's salt, (8) KO_2_, (9) Na_2_S, (10) Na_2_S_2_O_3_, (11) Na_2_SO_4_, (12) NaNO_2_, (13) HO˙, (14) OCl^–^, (15) ^*t*^BuOOH, (16) ^1^O_2_, (17) Na^+^, (18) Mg^2+^, (19) K^+^, (10) Ca^2+^, (21) Zn^2+^, (21) Fe^2+^, (23) blank. Error bars are ± S.D. All experiments were performed in 20 mM HEPES or 20 mM PBS (pH 7.4), containing <1% DMSO.

The selectivity of **PNCL** for ONOO^–^ was tested against a panel of biologically relevant reactive sulphur, oxygen, and nitrogen species and metal cations. The response of **PNCL** to 200 μM ONOO^–^ was compared against other analytes in HEPES buffer (20 mM, pH 7.4), by adding 5 mM reduced glutathione (GSH), 1 mM l-cysteine, or 200 μM other reactive species. Most of the species tested displayed no significant increase in luminescence intensity over the blank control within 20 min (Fig. 2). Given the destructive reactivity of HO˙, we examined the interference of this species by treating **PNCL** with ONOO^–^ in the presence of the HO˙-generating Fenton system (Fig. S4†), where only a minimal reduction in signal was observed. A small luminescence increase was observed when treating **PNCL** with Angeli's salt, commonly used as a nitroxyl (HNO) donor. This was consistent with previous observations using HPLC to track the reactivity of 5-fluoroisatin with Angeli's salt.^37^

**Fig. 3:**
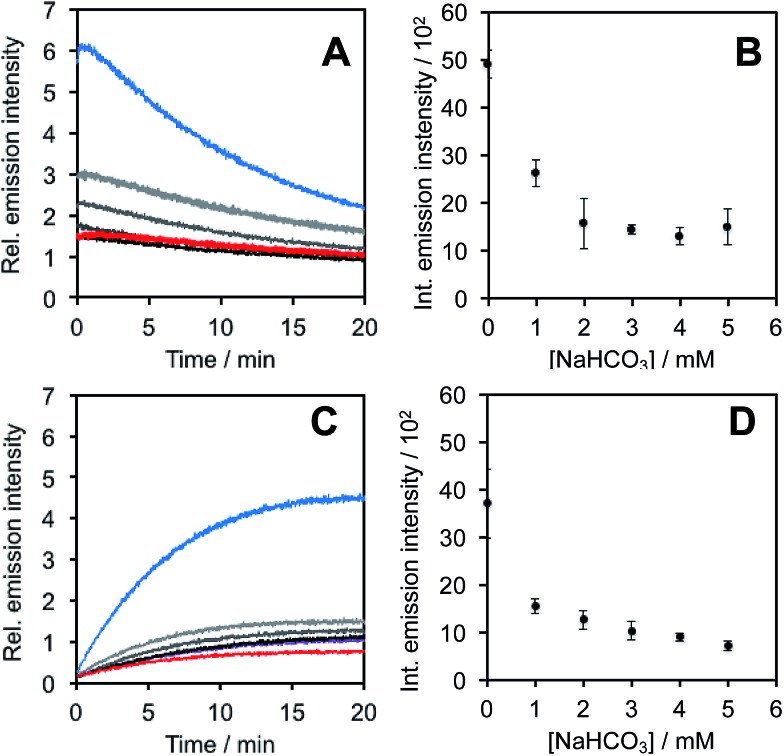
Inhibition of response by NaHCO_3_. (A) Time scans and (B) integrated intensity of the chemiluminescent emission of 20 μM **PNCL**, 200 μM ONOO^–^ and 0 (blue trace), 1 mM, 2 mM, 3 mM, 4 mM, and 5 mM (red trace) NaHCO_3_. (C) Time scans and (D) integrated intensity of the chemiluminescent emission of 20 μM **PNCL**, 200 μM Angeli's salt and 0 (blue trace), 1 mM, 2 mM, 3 mM, 4 mM, and 5 mM (red trace) NaHCO_3_. All experiments were performed in 20 mM HEPES (pH 7.4), containing <1% DMSO.

**Fig. 4:**
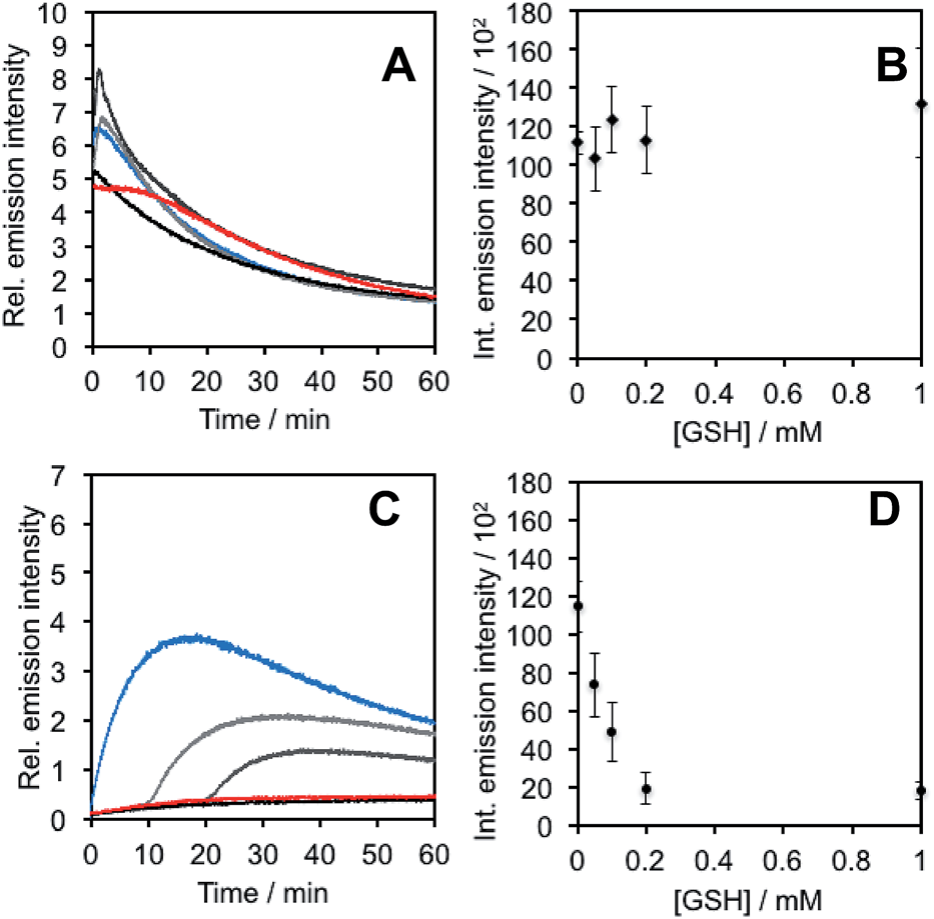
Inhibition of response by glutathione. (A) Time scans and (B) integrated intensity of the chemiluminescent emission of 20 μM **PNCL**, 200 μM ONOO^–^ and 0 (blue trace), 50 μM, 100 μM, 200 μM, and 1 mM (red trace) glutathione. (C) Time scans and (D) integrated intensity of the chemiluminescent emission of 20 μM **PNCL**, 200 μM Angeli's salt and 0 (blue trace), 50 μM, 100 μM, 200 μM, and 1 mM (red trace) glutathione. All experiments were performed in 20 mM HEPES (pH 7.4), containing <1% DMSO.

In light of studies exploring the generation of ONOO^–^ and other nitrogen oxide species from the autooxidation of NO^–^ and HNO,^17^–^19^ we performed a series of competition experiments with HCO_3_^–^ and GSH to better understand the luminescence increase resulting from reaction of **PNCL** with Angeli's salt. ONOO^–^ reacts rapidly with CO_2_, but slowly with GSH. HNO, on the other hand, reacts rapidly with GSH.^42^ We treated **PNCL** with ONOO^–^ (Fig. 3A and B) or Angeli's salt (Fig. 3C and D) in the presence of various amount of HCO_3_^–^ (1 mM, 2 mM, 3 mM, 4 mM, and 5 mM). HCO_3_^–^ decreased the chemiluminescence emission intensity generated by both ONOO^–^ and Angeli's salt, providing evidence that Angeli's salt can generate ONOO^–^. A similar experiment was performed by reacting **PNCL** (20 μM) with ONOO^–^ (Fig. 4A and B) or Angeli's salt (Fig. 4C and D) and various amounts of GSH (50 μM, 100 μM, 200 μM, and 1 mM). However, the GSH only quenched the luminescence intensity from Angeli's salt (Fig. 4B) and no significant luminescence decrease was observed from ONOO^–^ (Fig. 4A). Interestingly, substoichometric quantities of GSH would not only reduce the total amount of luminescence, but displayed a lag time before producing a chemiluminescent signal (Fig. 4C). Angeli's salt generates HNO with a half-life of 2.3 minutes at 37 °C. The observed lag time of the chemiluminescent response is consistent with GSH scavenging the newly formed HNO more rapidly than the autooxidation reaction of HNO. Once the GSH is consumed, HNO can be oxidized to ONOO^–^ or another nitrogen oxide species capable of two-electron chemistry and reaction with **PNCL** proceeds to produce a luminescent signal.

**Fig. 5:**
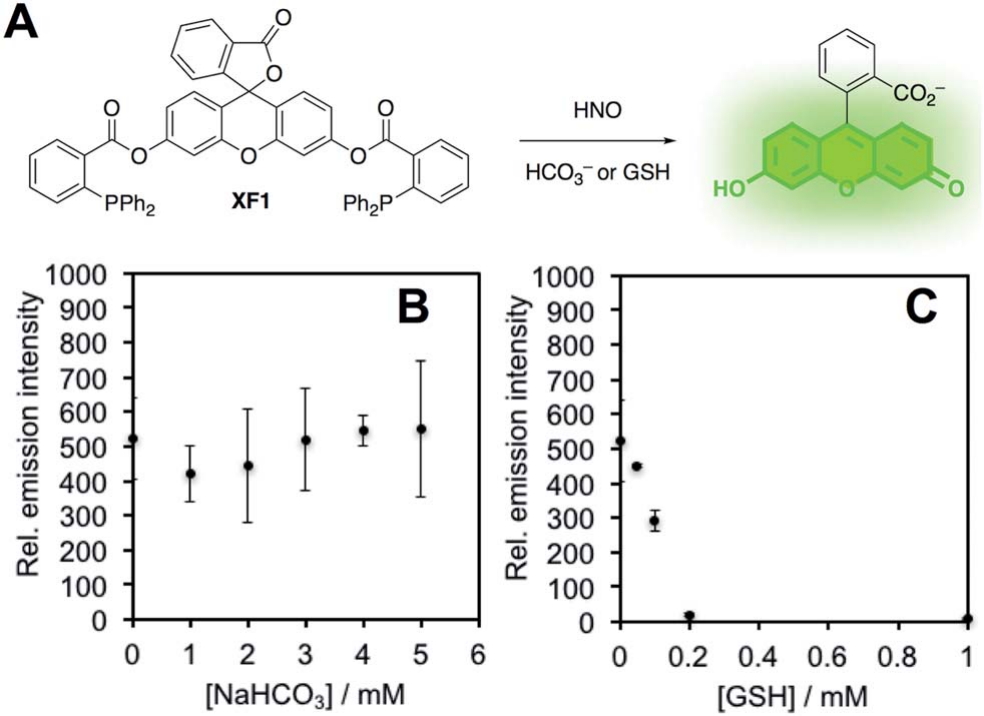
HNO scavenging by NaHCO_3_ and GSH. (A) Design of **XF1**, a fluorescent probe for HNO. (B) Peak emission intensity of 10 μM **XF1**, 200 μM Angeli's salt and 0–5 mM NaHCO_3_. (C) Peak emission intensity of 10 μM **XF1**, 200 μM Angeli's salt and 0–1 mM glutathione. All experiments were performed in 20 mM HEPES (pH 7.4), containing <1% DMSO with *λ*_ex_ = 488 nm.

In order to confirm this selectivity and that HCO_3_^–^/CO_2_ does not scavenge HNO, we prepared a fluorescent nitroxyl (HNO) probe, herein called **XF1** (Fig. 5A). HNO reacts with the diphenylphosphinobenzoyl group to form an aza-ylide that will nucleophilically attack the ester to trigger the release of the fluorophore.^43^,^44^ A time-dependent fluorescence turn-on was observed when treating 10 μM **XF1** with 200 μM Angeli's salt (Fig. S5A and B†), and selectivity tests demonstrated that **XF1** can distinguish HNO from other reactive sulfur, oxygen, and nitrogen species (Fig. S5C†). Inhibition experiments were conducted by treating **XF1** with various amounts of HCO_3_^–^ or GSH together with Angeli's salt. While no obvious inhibition of the fluorescence response was observed with the addition of HCO_3_^–^, the fluorescence was completely quenched with 200 μM GSH (Fig. 5B and C). These results confirm that GSH scavenges HNO generated from Angeli's salt but HCO_3_^–^/CO_2_ does not scavenge HNO. Taken together, the results from Fig. 3–5 are consistent with the luminescence increase from Angeli's salt being due to the generation of ONOO^–^. We do note that alternative oxidative species formed from HNO have been proposed.^18^ Although we cannot completely rule this out, the results here are consistent with the formation of ONOO^–^ from HNO and support the high specificity of **PNCL** for detecting ONOO^–^. As a final mechanistic investigation, we examined the selectivity of ONOO^–^*versus* NO_2_˙, HO˙, and CO_3_˙^–^ (the decomposition products of ONOO^–^) by treating isatin with ONOO^–^ in the presence of HCO_3_^–^/CO_2_ or TEMPOL, a free radical scavenger (Fig. S6†). While HCO_3_^–^/CO_2_ inhibits the reaction, TEMPOL has no effect. This suggests that isatin reacts directly with ONOO^–^ and not with free radical species like NO_2_˙, HO˙, or CO_3_˙^–^ that are formed *via* spontaneous ONOO^–^ decomposition.

### Evaluation of **PNCL** for ONOO^–^ detection in living cells

**Fig. 6:**
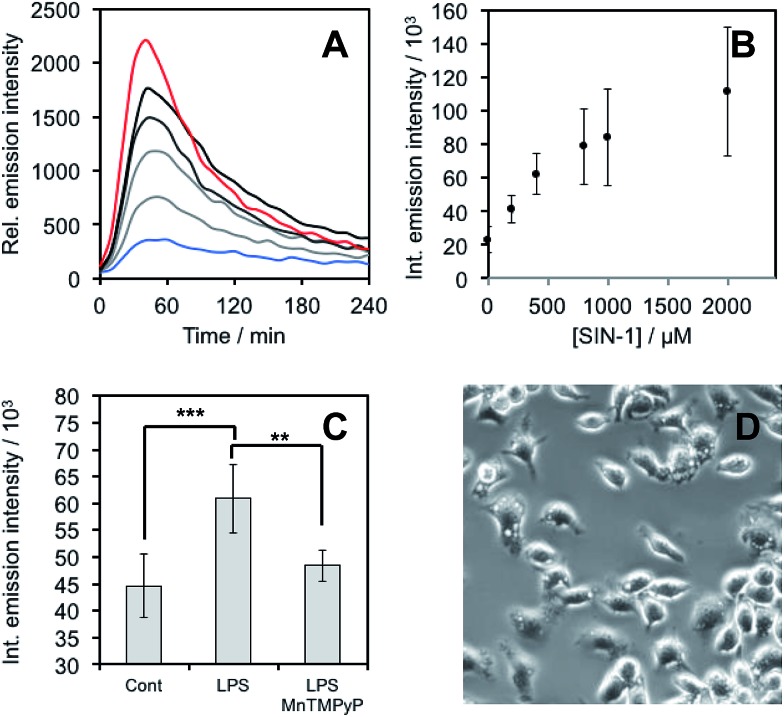
ONOO^–^ detection in RAW 264.7 macrophages. (A) Time scans and (B) integrated intensity of chemiluminescence emission of RAW 264.7 macrophages incubated with 20 μM **PNCL** for 30 minutes, washed, and then treated with 0 (blue trace), 200 μM, 400 μM, 800 μM, 1 mM, and 2 mM (red trace) SIN-1. (C) Integrated chemiluminescent emission intensity of RAW 264.7 macrophages stimulated with (Cont) Vehicle control, (LPS) 1000 ng mL^–1^ LPS, or (LPS, MnTMPyP) 1000 ng mL^–1^ LPS and 25 μM Mn(iii)TMPyP and then treated with 20 μM **PNCL**. (D) Brightfield images of stimulated RAW 264.7 macrophages. Error bars are S.D. from *n* = 3–7 wells and 3 biological replicates (*n* = 10 wells from 6 biological replicates for the control experiment). Statistical significance was assessed using a two-tailed student's *t*-test. ***p < 0.001, **p < 0.01.

After demonstrating that **PNCL** reacts with ONOO^–^ with high sensitivity and selectivity, we then proceeded to investigate the ability of **PNCL** for ONOO^–^ detection in living cells using a BioTek Cytation 5 plate reader. Toxicity was determined by the MTT assay, which indicated low toxicity after 24 hour treatment with **PNCL** at concentrations less than 100 μM and more than 50% viability after treating with high concentrations of 1 mM **PNCL** for 24 hours (Fig. S7†). Cellular internalization was verified by imaging the intrinsic fluorescence emission of **PNCL** (Fig. S8†). In order to evaluate the ability of **PNCL** to detect cellular delivery of ONOO^–^ from donor compounds, RAW 264.7 macrophages were incubated with **PNCL** for 30 minutes, washed, and then treated with 0, 200 μM, 400 μM, 800 μM, 1 mM, or 2 mM SIN-1, a commonly used ONOO^–^ donor that operates by simultaneously generating NO˙ and O_2_˙^–^. A time course of the chemiluminescent emission intensity increased to reach a maximum at 40 minutes (Fig. 6A). Both the peak and integrated luminescence intensities increased with increasing concentrations of SIN-1 (Fig. 6A and B), and a 5-fold luminescence increase was obtained with 2 mM SIN-1 compared with a blank control. We also performed the same experiment in A549 cells (Fig. S9†). Similar results were observed and excellent linearity was achieved in living cells between 0 and 400 μM SIN-1 (Fig. S9C†). This increase in chemiluminescent emission from SIN-1 and **PNCL** was attenuated by the ONOO^–^ scavenger Mn(iii)TMPyP (Fig. S10†), confirming that the response is due to ONOO^–^.

Finally, we investigated the ability of **PNCL** to detect ONOO^–^ generated by macrophages stimulated to mount an immune response. Lipopolysaccharide (LPS) binds to the toll-like receptors of macrophages, activating the transcription factor NF-*κ*β ultimately leading to the expression of iNOS and generation of reactive oxygen species that can produce ONOO^–^.^12^ Macrophages stimulated with LPS produced increased chemiluminescent signal from **PNCL** (Fig. 6C), showing a 36% increase in integrated luminescence intensity with 1000 ng mL^–1^ LPS. Treatment with the ONOO^–^ scavenger Mn(iii)TMPyP reduces this signal back to control levels and co-incubation with the selective iNOS inhibitor 1400 W also reduces the signal from LPS and **PNCL** (Fig. S11†). These control experiments validate the ability of **PNCL** to detect cellular production of ONOO^–^. Bright field images of the cells after LPS stimulation show the formation of phagosomes (Fig. 6D), a phenotypic response to LPS exposure.

## Conclusions

We have designed and synthesized a reaction-based chemiluminescent probe for ONOO^–^, **PNCL**. **PNCL** reacts with ONOO^–^ through an oxidative decarbonylation reaction to initiate light emission that can be observed instantly with high sensitivity and selectivity against other reactive sulphur, oxygen, and nitrogen species. The observed reactivity towards Angeli's salt is attributed to the generation of ONOO^–^, which is supported by inhibitor experiments with HCO_3_^–^ and GSH, providing experimental evidence of an autooxidative product of HNO. Several studies have purported that the autooxidation of HNO produces a product unique from ONOO^–^, as evidenced by data showing no effect of HCO_3_^–^ on the reaction between Angeli's salt and reactive oxygen species probe dihydrorhodamine 123.^18^ In our studies, however, we observe a drastic HCO_3_^–^ dependent reduction in chemiluminescent signal from **PNCL** and Angeli's salt, consistent with ONOO^–^ formation from HNO under physiological conditions.^19^**PNCL** was used to detect ONOO^–^ generated from donor compounds in multiple cell lines, as well as cellular ONOO^–^ generated by macrophages stimulated with LPS. Confirmation of the chemical specificity towards ONOO^–^ over downstream free radical products, coupled with operational compatibility in living cells establishes **PNCL** a useful and easy-to-use tool for studying ONOO^–^ in chemical and biological systems.

## Conflicts of interest

A. R. L. discloses a financial stake in BioLum Science, LLC, a company developing asthma monitoring technology.

## Supplementary Material

Supplementary information
