# Supplementary material for: A chemiluminescent probe for cellular peroxynitrite using a self-immolative oxidative decarbonylation reaction
Source: Chem Sci. 2018 Jan 31;9(9):2552–8. doi: 10.1039/c7sc05087a (PMC5914148; doi:10.1039/c7sc05087a)
Supplement: Supplementary file 1 [file SC-009-C7SC05087A-s001.pdf]

Electronic Supplementary Material (ESI) for Chemical Science.
This journal is © The Royal Society of Chemistry 2018

Supporting Information:

A Chemiluminescent Probe for Cellular Peroxynitrite Using an Oxidative Decarbonylation Reaction

Jian Cao,[†,§] Weiwei An,[†,§] Audrey G. Reeves,[†] and Alexander R. Lippert*[†,§,¶]

[†]Department of Chemistry, [§]Center for Drug Discovery, Design, and Delivery (CD4), and [¶]Center for Global Health Impact (CGHI), Southern Methodist University, Dallas, TX, 75275-0314.

Table of contents Pages **Pages**

Section	Pages
1. General methods for chemical synthesis	S2
2. Preparation of $ONOO^-$	S4
3. GC-MS experiment to monitor reaction intermediates	S4
4. Chemiluminescence response	S5
5. Determination of the detection limit of bolus $ONOO^-$	S6
6. Selectivity tests	S7
7. Synthesis of **XF1**	S8
8. **XF1** response and selectivity tests	S9
9. Inhibition experiments with HCO_3^- and TEMPOL	S10
10. Inhibition experiments from HCO_3^- and glutathione	S10
11. Cell culture	S11
12. MTT assay	S12
13. Cellular internalization of **PNCL**	S13
14. Detection of SIN-1 generated $ONOO^-$ in macrophages	S13
15. Application of **PNCL** for detecting endogenous $ONOO^-$	S15
16. iNOS inhibit the production of $ONOO^-$ in macrophages	S15
17. ^1H and ^{13}C NMR Spectra	S17

1. General Methods for Chemical Synthesis. All reactions were performed in dried glassware under an atmosphere of dry N_2. Silica gel P60 (SiliCycle) was used for column chromatography

and SiliCycle 60 F254 silica gel (precoated sheets, 0.25 mm thick) was used for analytical thin layer chromatography. Plates were visualized by fluorescence quenching under UV light or by staining with iodine. Other reagents were purchased from Sigma-Aldrich (St. Louis, MO), Alfa Aesar (Ward Hill, MA), EMD Millipore (Billerica, MA), Oakwood Chemical (West Columbia, SC), and Cayman Chemical (Ann Arbor, MI) and used without further purification. ^{1}H NMR and ^{13}C NMR spectra for characterization of new compounds and monitoring reactions were collected in $CDCl_3$ (Cambridge Isotope Laboratories, Cambridge, MA) on a JEOL 500 MHz spectrometer in the Department of Chemistry at Southern Methodist University. All chemical shifts are reported in the standard notation of parts per million using the peak of residual proton signals of the deuterated solvent as an internal reference. Coupling constant units are in Hertz (Hz) Splitting patterns are indicated as follows: br, broad; s, singlet; d, doublet; t, triplet; q, quartet; m, multiplet; dd, doublet of doublets; dt, doublet of triplets. High-resolution mass spectroscopy was performed on a Shimadzu IT-TOF (ESI source) at the Shimadzu Center for Advanced Analytical Chemistry at the University of Texas, Arlington and Matrix-assisted desorption/ionization (MALDI) mass spectrometry was performed at the University of Texas, Dallas.

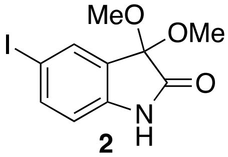

2

5-iodo-3,3-dimethoxyindolin-2-one (2). 5-iodoisatin (203.1 mg, 7.43 mmol, 1.0 equiv) was dissolved in 300 mL MeOH, followed directly by the addition of 7.1 mL HCl. The reaction was stirred for 48 h at rt. The reaction mixture was neutralized with saturated $NaHCO_3$, and then the reaction mixture was poured into a separatory funnel containing 150 mL H_2O, and extracted with 3 x 100 mL EtOAc. The organic layer was washed with 10 mL brine, dried over Na_2SO_4, filtered, and concentrated. Purification by silica column chromatography (1:4 EtOAc/hexanes) afforded **2** (2.2 g, 93%) as a colorless solid. ^{1}H NMR (500 MHz, $CDCl_3$) δ 9.46 (s, 1H), 7.65 (d, 1H, J = 1.7 Hz), 7.58 (dd, 1H, J = 8.0, 1.7 Hz), 6.70 (d, 1H, J = 8.0 Hz), 3.53 (s, 6H); ^{13}C NMR (125 MHz, $CDCl_3$) δ 173.02, 140.40, 139.61, 133.84, 127.42, 113.36, 97.42, 85.43, 51.08. HRMS calcd for $C_{10}H_{10}INO_3$ (M–H^{-}) 317.9633, found 317.9638.

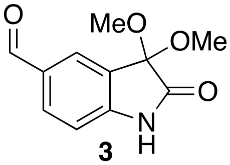

3

3,3-dimethoxy-2-oxoindoline-5-carbaldehyde (3). Compound **2** (607.0 mg, 1.90 mmol, 1.0 equiv), *N*-formyl saccharin (602.8 mg, 2.86 mmol, 1.5 equiv), palladium(II) acetate (12.8 mg, 0.057 mmol, 0.03 equiv), 1,4-bis(diphenylphosphino)butane (36.5 mg, 0.086 mmol, 0.045 equiv), and Na_2CO_3 (302.3 mg, 2.86 mmol, 1.5 equiv) were added to a 10 mL pressure flask. The flask was evacuated and backfilled with N_2 three times. Then, a degassed solution of Et_3SiH (395 µL, 2.47 mmol, 1.3 equiv) and anhydrous DMF (2 mL) were added to the flask under N_2. The pressure flask was sealed immediately and the mixture was stirred for 10 min at rt. Then the mixture was warmed to 80 °C and stirred for 16 h. The reaction was quenched with 100 mL saturated NH_4Cl, extracted with 3 x 80 mL EtOAc, washed with 10 mL brine, dried over Na_2SO_4, filtered, and concentrated. The crude was purified by the silica column chromatography (1:3 EtOAc/hexanes) to deliver compound **4** as a white solid (245.0 mg, 59%). ^{1}H NMR (500 MHz, $CDCl_3$) δ 9.90 (s,

1H), 9.33 (s, 1H), 7.93 (d, 1H, *J* = 1.7 Hz), 7.87 (dd, 1H, *J* = 8.0, 1.7 Hz), 7.07 (d, 1H, *J* = 8.0 Hz), 3.59 (s, 6H); ^{13}C NMR (125 MHz, $CDCl_3$) δ 190.81, 173.33, 146.20, 134.69, 131.82, 126.25, 125.85, 111.36, 96.60, 51.02. HRMS calcd for $C_{11}H_{11}NO_4$ ($M–H^-$) 220.0615, found 220.0609.

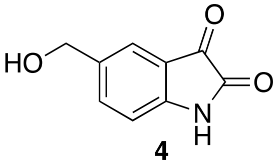

4

5-(hydroxymethyl)indoline-2,3-dione (4). Compound **3** (160.0 mg, 0.72 mmol, 1.0 equiv) and sodium borohydride (41.9 mg, 1.11 mmol, 1.53 equiv) were dissolved in 12 mL EtOH, and the mixture was stirred at rt for 1 h. The reaction was quenched with 80 mL saturated NH_4Cl, extracted with 3 x 40 mL EtOAc, washed with 10 mL brine, dried over Na_2SO_4, filtered, and concentrated to yield 5-(hydroxymethyl)-3,3-dimethoxyindolin-2-one (162 mg) as a colorless oil and used without further purification. ^{1}H NMR (500 MHz, $CDCl_3$) δ 9.05 (s, 1H), 7.39 (s, 1H), 7.24 (d, 1H, *J* = 8.0 Hz), 6.79 (d, 1H, *J* = 8.0 Hz), 4.61 (s, 2H), 3.53 (s, 6H); ^{13}C NMR (125 MHz, $CDCl_3$) δ 173.33, 140.14, 135.67, 129.81, 125.43, 124.28, 111.05, 97.41, 64.88, 50.95. HRMS calcd for $C_{11}H_{13}NO_4$ ($M–H^-$) 222.0772, found 222.0778. 5-(hydroxymethyl)-3,3-dimethoxyindolin-2-one (189 mg, 0.85 mmol, 1.0 equiv) was dissolved in 12 mL 1 M HCl solution, and the reaction was stirred at rt for 30 min. The reaction was neutralized with saturated $NaHCO_3$, and then the reaction mixture was poured into a separatory funnel, and extracted with 3 x 40 mL 3:1 DCM: iPrOH and 3 x 30 mL EtOAc. The organic layer was washed with 10 mL brine, dried over Na_2SO_4, filtered, and concentrated. Purification by silica column chromatography (1:1 to 2:1 EtOAc/hexanes) afforded **4** (132.0 mg, 88%) as an orange solid. ^{1}H NMR (500 MHz, $(CD_3)_2CO$) δ 7.58 (d, 1H, *J* = 8.0 Hz), 7.50 (dd, 1H, *J* = 8.0, 1.7 Hz), 6.96 (d, 1H, 1.7 Hz), 4.57 (s, 2H); ^{13}C NMR (125 MHz, $(CD_3)_2CO$) δ 184.09, 159.14, 149.60, 137.82, 136.77, 122.77, 118.10, 112.00, 62.81. HRMS calcd for $C_9H_7NO_3$ ($M–H^-$) 176.0353, found 176.0345.

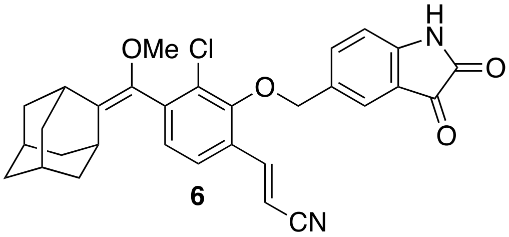

6

(*E*)-3-(4-(((1*r*,3*r*,5*R*,7*S*)-adamantan-2-ylidene)(methoxy)methyl)-3-chloro-2-((2,3-dioxoindolin-5-yl)methoxy)phenyl)acrylonitrile (6). Compound **4** (20 mg, 0.11 mmol, 1.0 equiv), compound **5**[1] (43.8 mg, 0.11, 1.0 equiv), and triphenylphosphine (36.0 mg, 0.13 mmol, 1.2 equiv) were dissolved in 2 mL anhydrous THF, and the reaction mixture was cooled to 0 °C. Diethyl azodicarboxylate (21.3 µL, 0.14 mmol, 1.2 equiv) was added dropwise over 5 min. After 1 h of stirring at rt, the mixture was concentrated. Purification by silica column chromatography (2% MeOH/DCM) afforded **6** as a yellow oil (49.0 mg, 84%). ^{1}H NMR (500 MHz, $CDCl_3$) δ 8.56 (s, 1H), 7.71 (dd, 1H, *J* = 8.0, 1.7 Hz), 7.64 (d, 1H, *J* = 1.7 Hz), 7.50 (d, 1H, *J* = 16.8 Hz), 7.12 (d, 1H, *J* = 8.0 Hz), 7.02 (d, 1H, *J* = 8.0 Hz), 5.90 (d, 1H, *J* = 16.8 Hz), 4.98 (m, 2H), 3.32 (s, 3H), 3.27 (s, 1H), 1.64-2.20 (m, 13 H); ^{13}C NMR (125 MHz, $CDCl_3$) δ 182.76, 159.24, 152.97, 149.62,

---

1. O. Green, T. Eilon, N. Hananya, S. Gutkin, C. R. Bauer and D. Shabat, *ACS Cent. Sci*. 2017, **3**, 349.

144.73, 139.66, 139.05, 133.50, 131.91, 129.98, 128.40, 125.88, 124.59, 118.24, 117.98, 112.85, 98.63, 75.11, 57.56, 39.27, 39.12, 38.73, 37.05, 33.11, 29.85, 29.36, 28.38, 28.21. HRMS calcd for $C_{30}H_{27}ClN_2O_4$ (M–H⁻) 513.1587, found 513.1595.

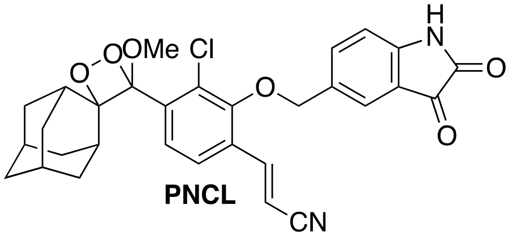

PNCL

(*E*)-3-(3-chloro-2-((2,3-dioxoindolin-5-yl)methoxy)-4-((1*r*,3*r*,5*r*,7*r*)-4'-methoxyspiro[adamantane-2,3'-[1,2]dioxetan]-4'-yl)phenyl)acrylonitrile (PNCL). Enol ether **6** (40.0 mg, 0.075 mmol, 1.0 equiv) and Rose bengal (9.8 mg, 0.010 mmol, 0.13 equiv) were added into a dry flask and dissolved in 5 mL THF. Oxygen was bubbled through the reaction mixture, while irradiating with a 120 W light bulb (Home Depot, Dallas, TX) at 0 °C. After 3 h of reaction, TLC showed no starting material left and the mixture was then concentrated under vacuum at 0 °C and the residue was purified by the silica column chromatography (10:1 DCM/Ether) to deliver PNCL (33.0 mg, 81%) as a yellow solid. ^{1}H NMR (500 MHz, $CDCl_3$) δ 9.02 (s, 1H), 7.97 (d, 1H, *J* = 8.6 Hz), 7.66-7.70 (m, 2H), 7.25-7.52 (m, 2H), 7.12 (d, 1H, *J* = 8.0 Hz), 7.04 (d, 1H, *J* = 8.0 Hz), 5.96 (d, 1H, *J* = 16.6 Hz), 4.90 (s, 2H), 3.22 (s, 3H), 3.02 (s, 1H), 1.24-2.20 (m, 13H); ^{13}C NMR (125 MHz, $CDCl_3$) δ 182.77, 159.21, 153.45, 149.72, 144.23, 138.94, 136.76, 131.60, 120.50, 129.62, 125.86, 124.77, 118.30, 117.65, 112.92, 111.63, 100.03, 96.49, 75.26, 49.87, 36.58, 33.98, 33.72, 32.79, 32.23, 31.64, 31.05, 29.79, 26.18, 25.87. MALDI-MS calcd for $C_{30}H_{27}ClN_2O_6$ (M+H⁺) 547.16, found 547.15.

2. Preparation of ONOO⁻.[2] To a 200 mL round flask was added 2.2 mL of 11.6 M H_2O_2 (26.0 mmol) further diluted with 50 mL DI-H_2O. The mixture was allowed to chill to 0 °C before addition of 36 mL of 0.55 M NaOH followed by 5 mL of 0.04 M EDTA. The final mixture was diluted to a total volume of 100 mL with DI-H_2O before the addition of 3.4 mL isopentyl nitrite (26.0 mmol). A deep, yellow color indicates the formation of peroxynitrite. After 6 hours of stirring at 0 °C, the mixture was washed 3 x 20 mL with DCM. To the aqueous layer was added roughly 20 mg of MnO_2. The mixture was allowed to stir until bubbling ceased, upon which it was filtered twice and stored on ice or in the freezer. The concentration was determined using an extinction coefficient of $\varepsilon = 1670\ M^{-1}\ cm^{-1}$ at 302 nm.

3. GC-MS procedure to monitor reaction intermediates

300 µL of 10 mM **PNCL** in DMSO (3 mM final concentration), and 125 µL of 48 mM ONOO⁻ (6 mM final concentration) were mixed in an Eppendorf tube, and vortexed for 5 second. The reaction mixture was then poured into a separatory funnel containing 10 mL EtOAc and 15 mL DI-H_2O and extracted with 2 x 10 mL EtOAc. The organic layer was collected, dried over Na_2SO_4, filtered, and concentrated. Then the solid was re-dissolved in 2 mL CH_2Cl_2, transferred to a GC-MS vial and GC-MS was conducted immediately.

---

2. R. M. Uppu and W. A. Pryor, *Anal. Biochem*. 1996, **236**, 242.

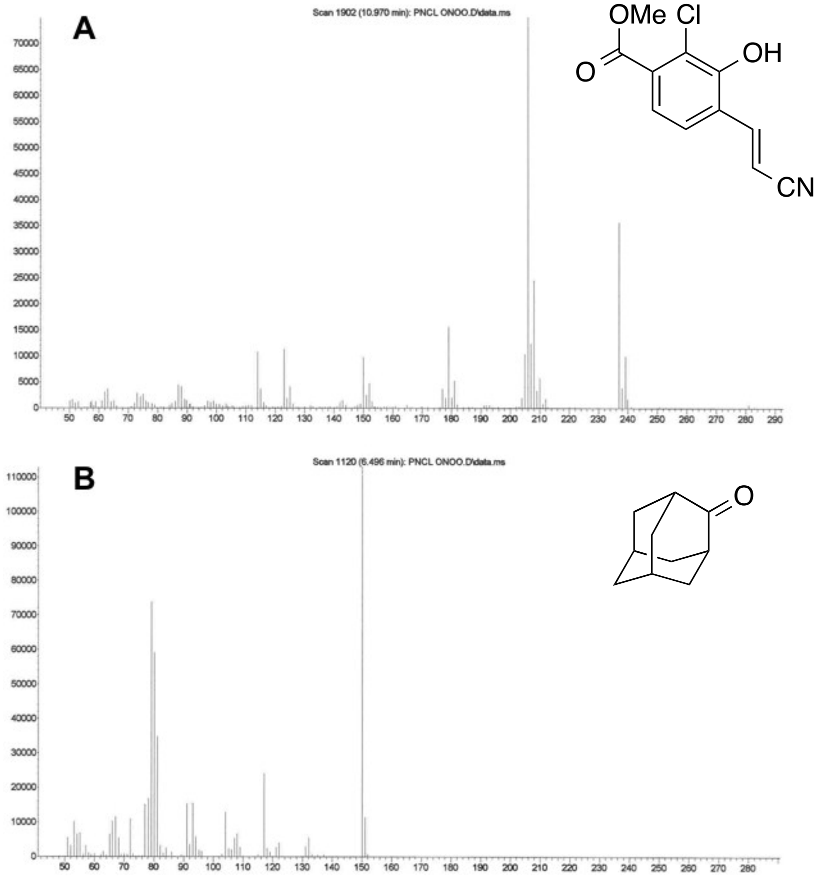

Figure S1. The reaction products were observed between **PNCL** and $ONOO^-$ by GC-MS. (A) m/z=237, (B) m/z=150.1.

4. Chemiluminescent response. Chemiluminescent responses and time scans were acquired with a Hitachi F-7000 Fluorescence Spectrophotometer (Hitachi, Tokyo, Japan) using the luminescence detection module. 20 mM HEPES buffer (pH 7.4), 5 mM **PNCL** stock solution in DMSO and $ONOO^-$ were added to a quartz cuvette (Starna, Atascadero, CA) in sequences. Samples were shaken gently to assure mixing. 20 μM **PNCL** was treated with 0, 5, 10, 20, 40, 80, 100 and 200 μM $ONOO^-$. Time scans were acquired using the time scan module by setting the emission wavelength to 525 nm. Wavelength scans were acquired by treating 20 μM **PNCL** with 0 μM to 200 μM $ONOO^-$. Time scans and wavelength scans were measured 10 second after adding $ONOO^-$.

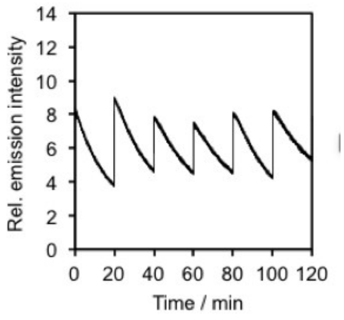

Figure S2. Repeatability of chemiluminescence response. Time course of the chemiluminescence emission at 525 nm of 20 μM **PNCL** and 200 μM $ONOO^{-}$ in 20 mM HEPES (pH 7.4). Every 20 min, an additional 200 μM $ONOO^{-}$ was added to the solution.

5. Determination of the detection limit of bolus $ONOO^{-}$

Chemiluminescent responses were measured using a Cytation 5 BioTek plate reader (Winooski, VT) by using the luminescence detection method, endpoint read type, and setting gain to 135 and temperature to 37 °C. 0.125 mM, 0.25 mM, 0.5 mM, 0.75 mM $ONOO^{-}$ stock solutions were made by dilution of a 16 mM $ONOO^{-}$ solution with 0.01 M NaOH, and the final concentration was also confirmed by UV/Vis. 249 μL of 10 mM PBS buffer (pH 7.4) was added into vehicle well, and 248 μL of 20 mM HEPES buffer (pH 7.4) was added into other wells. 1 μL of 5 mM **PNCL** was pipetted into every well, and then 1 μL of different concentrated $ONOO^{-}$ stock solutions were added into wells. The chemiluminescence emission was then measured on the plate reader. The emission was integrated over 30 minutes and the calibration curve was constructed using three replicate experiments. The detection limit was determined as LoD = 3σ/k.

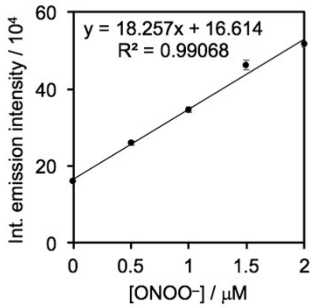

Figure S3. Detection Limit of **PNCL** for bolus $ONOO^{-}$. Integrated chemiluminescence emission intensity of 20 μM **PNCL** and 0 μM, 0.5 μM, 1 μM, 1.5 μM, and 2 μM of $ONOO^{-}$.

Table S1. Detection limits of selected fluorescent and chemiluminescent $ONOO^-$ probes.

Reference	Method	LoD
3	Fluorescence	2.5 µM
4	Fluorescence	0.917 µM
5	Chemiluminescence	0.1 µM
6	Fluorescence	50 nM
7	Fluorescence	35 nM
8	Fluorescence	10 nM
this work	Chemiluminescence	6 nM
9	Chemiluminescence	5 nM
10	Fluorescence	4 nM

6. Selectivity tests

Selectivity for **PNCL** was measured by monitoring the time-dependent chemiluminescent emission at 525 nm with an F-7000 fluorescence spectrophotometer (Hitachi, Tokyo, Japan). All assays were performed in 20 mM HEPES buffered to pH 7.42. All analytes were tested with final concentration of 200 µM, with the exceptions of glutathione (5 mM), and L-cysteine (1 mM).

$ONOO^-$ (200 µM): 3.1 µL of 65 mM $ONOO^-$ was added to a solution of 993 µL HEPES buffer and then 4 µL of 5 mM **PNCL** in DMSO was added to the mixture.

$S_2O_3^{2-}$ (200 µM): 4 µL of 50 mM $Na_2S_2O_3$ in DI-H_2O was added to a solution of 992 µL HEPES buffer and then 4 µL of 5 mM **PNCL** in DMSO was added to the mixture.

^{t}BuOOH (200 µM): 4 µL of 50 mM ^{t}BuOOH in DI-H_2O was added to a solution of 992 µL HEPES buffer and then 4 µL of 5 mM **PNCL** in DMSO was added to the mixture.

S-nitrosoglutathione (200 µM): 20 µL of 10 mM *S*-nitrosoglutathione in DI-H_2O was added to a solution of 976 µL HEPES buffer and then 4 µL of 5 mM **PNCL** in DMSO was added to the mixture.

Glutathione (5 mM): 20 µL of 250 mM glutathione in DI-H_2O was added to a solution of 976 µL HEPES buffer and then 4 µL of 5 mM **PNCL** in DMSO was added to the mixture.

L-Cysteine (1 mM): 4 µL of 250 mM cysteine in DI-H_2O was added to a solution of 992 µL HEPES buffer and then 4 µL of 5 mM **PNCL** in DMSO was added to the mixture.

---

3. J. Kim, J. Park, H. Lee, Y. Choi and Y. Kim, *Chem. Comm.* 2014, **50**, 9353.
4. F. Yu, P. Li, B. Wang, and K. Han, J. Am. Chem. Soc. 2013, **135**, 7674.
5. W. Zhou, Y. Cao, D. Sui and C. Lu, *Anal. Chem.* 2016, **88**, 2659.
6. T. Peng and D. Yang, *Org. Lett.* 2010, **12**, 4932.
7. J. Li, C. S. Lim, G. Kim, H. M. Kim and J. Yoon, *Anal. Chem*. 2017, **89**, 8496.
8. T. Peng, N. K. Wong, X. Chen, Y. K. Chan, D. H. H. Ho, Z. Sun, J. J. Hu, J. Shen, H. El-Nezami and D. Yang, J. Am. Chem. Soc. 2014, **136**, 11728.
9. W. Zhou, S. Dong, Y. Lin, C. Lu, *Chem. Commun.* 2017, **53**, 2122.
10. H. Zhang, J. Liu, Y. Q. Sun, Y. Huo, Y. Li, W. Liu, X. Wu, N. Zhu, Y. Shi and W. Guo, *Chem. Commun*. 2015, **51**, 2721.

NaNO$_2$ (200 μM): 4 μL of 50 mM NaNO$_2$ in DI-H$_2$O was added to a solution of 992 μL HEPES buffer and then 4 μL of 5 mM **PNCL** in DMSO was added to the mixture.

Na$_2$SO$_4$ (200 μM): 4 μL of 50 mM Na$_2$SO$_4$ in DI-H$_2$O was added to a solution of 992 μL HEPES buffer and then 4 μL of 5 mM **PNCL** in DMSO was added to the mixture.

Na$_2$S (200 μM): 4 μL of 50 mM Na$_2$S in DI-H$_2$O was added to a solution of 992 μL HEPES buffer and then 4 μL of 5 mM **PNCL** in DMSO was added to the mixture.

H$_2$O$_2$ (200 μM): 4 μL of 50 mM H$_2$O$_2$ in DI-H$_2$O was added to a solution of 992 μL HEPES buffer and then 4 μL of 5 mM **PNCL** in DMSO was added to the mixture.

NO$^•$ (200 μM): DEA NONOate was used to generate NO. It was stored at –20 °C and dissolved in 0.01 M NaOH solution immediately prior to use. The concentration of this alkaline stock solution of DEA NONOate was measured by UV/Vis using ε = 6500 M^{-1} cm^{-1} at 250 nm. 6.36 μL of 31.45 mM DEA NONOate in 0.01 M NaOH solution was added to a 994 μL solution of 20 μM **PNCL** in HEPES buffer.

HNO (200 μM): Angeli's salt (Na$_2$N$_2$O$_3$) was used to generate HNO. The stock solution was made by dissolving Angeli's salt in 0.01 M NaOH solution immediately prior to use. The concentration of this alkaline stock solution of Angeli's salt was measured by UV/Vis is using ε = 6100 M^{-1} cm^{-1} at 237 nm. 6.33 μL of 31.55 mM stock solution of H$_2$O$_2$ in DI-water was added to a 994 μL solution of 20 μM **PNCL** in HEPES buffer.

O$_2^{•-}$ (200 μM): 1 mg KO$_2$ (final concentration 200 μM) was added to a 70 mL solution of 20 μM **PNCL** in HEPES buffer.

OH$^•$ (200 μM): 1 mg Fe(ClO$_4$)$_2$ (final concentration 200 μM) was added to a 30 mL solution of 20 μM **PNCL** and H$_2$O$_2$ (final concentration 200 μM) in HEPES buffer.

OCl$^-$ (200 μM): 4 μL of 50 mM NaOCl in DI-H$_2$O was added to a solution of 992 μL HEPES and 4 μL of 5 mM **PNCL** in DMSO was added into this mixture.

$^1O_2$: 4 μL of 5 mM **PNCL** in DMSO, 8 μL of 25 mM Rose bengal in DI-H$_2$O were added to a solution of 988 μL HEPES and illuminated with 500 nm wavelength for 10 min before time scanning. Longer illumination times of 30 minutes also showed no response.

Blank: 3.1 μL of a 0.20 M NaOH in DI-H$_2$O was added to a solution of 993 μL HEPES and 4 μL of 5 mM **PNCL** in DMSO was added into this mixture.

OH$^•$ interference: 4 μL of 50 mM FeCl$_2$ and 4 μL of 50 mM H$_2$O$_2$ were added to a solution of 984 μL HEPES buffer containing 20 μM **PNCL**. 4 μL of 50 mM ONOO$^-$ was then added to that solution, and the mixture was shaken gently before testing.

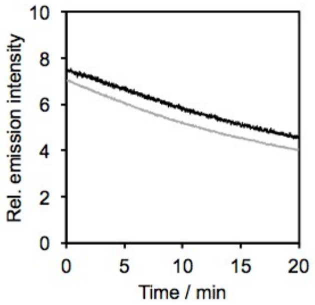

Figure S4. Chemiluminescence response of 20 μM **PNCL** and 200 μM ONOO⁻ in the (grey trace) presence of OH$^{\bullet}$ and (black trace) absence of OH$^{\bullet}$. Luminescence responses were collected using the time scan module by setting the emission wavelength to 525 nm.

7. Synthesis of XF1

3 - Oxo - 3H - spiro [isobenzofuran - 1, 9' - xanthene]- 3', 6' - diyl bis (2 - (diphenylphosphanyl)benzoate) (XF1). 2-(diphenylphosphino) benzoic acid (336.9 mg, 1.1 mmol, 2.2 equiv) and HBTU (417.2 mg, 1.1 mmol, 2.2 equiv) were dissolved in 5 mL of DMF. DIPEA (0.52 mL, 3.0 mmol, 6.0 equiv) was added and the reaction was stirred for five minutes. Fluorescein (166.2 mg, 0.5 mmol, 1.0 equiv) was added and the reaction was stirred for 24 h. The reaction mixture was concentrated and purified by silica gel column chromatography (1:1 Hexanes/EtOAc) to yield **XF1** (78.4 mg, 17% yield) as a white solid. ^{1}H NMR (500 MHz, $CDCl_3$) δ 8.25–8.23 (m, 2H), 8.01 (d, 1H, J = 7.5 Hz), 7.66 (td, 1H, J = 7.5, J = 1.2 Hz), 7.61 (td, 1H, J = 7.5, J = 1.2 Hz), 7.49–7.45 (m, 4H), 7.35–7.25 (m, 20H), 7.12 (d, 1H, J = 7.4 Hz), 7.01–6.98 (m, 2H), 6.90 (d, 2H, J = 2.3 Hz), 6.74 (d, 2H, J = 8.6 Hz), 6.63 (dd, 2H, J = 8.6 Hz, 2.3 Hz); ^{13}C NMR (125 MHz, $CDCl_3$) δ 169.53, 164.86, 153.29, 151.93, 151.48, 137.53, 137.45, 135.35, 134.56, 134.20, 134.04, 133.08, 132.93, 132.86, 131.92, 131.52, 130.06, 129.28, 128.97, 128.89, 128.74, 128.68, 128.45, 126.07, 125.23, 124.24, 117.85, 116.43, 110.59, 81.85; HRMS calcd for $C_{58}H_{38}O_7P_2$ $[M+Na]^+$ 931.1985, found 931.1990.

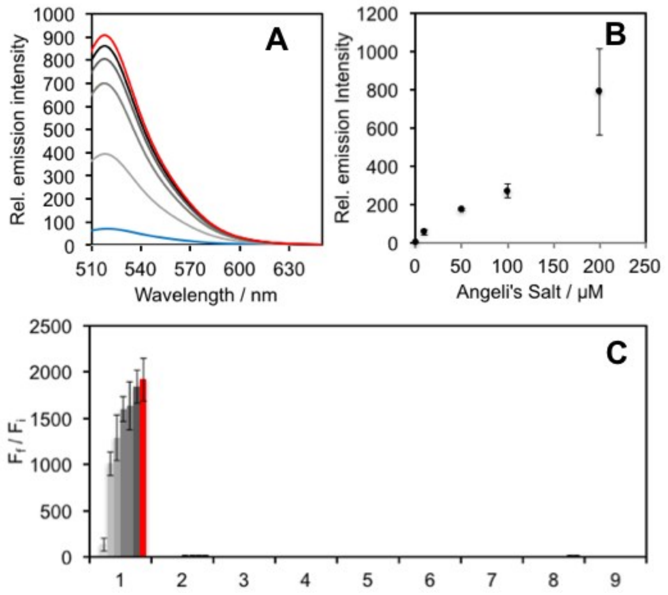

Figure S5. (A) Fluorescence emission of 10 μM **XF1** and 200 μM Angeli's salt after reacting for 1, 5, 10, 15, 20, 25, and 30 min in 20 mM HEPES (pH 7.42). (B) Peak emission intensity of 10 μM **XF1** and 0, 5, 50, 100, and 200 μM Angeli's salt after reacting for 30 min in 20 mM HEPES (pH 7.4). (C) Selectivity of **XF1** versus other reactive sulfur, oxygen, and nitrogen species. Legend: 1. Angeli's salt, 2. Na_2S, 3. $ONOO^-$, 4. ClO^-, 5. H_2O_2, 6. GSH (5 mM), 7. GSNO, 8. DEA NONOate, 9. ^tBuOOH.

8. XF1 response and selectivity tests

Response. Wavelength scan of fluorescent emission of 10 μM **XF1** at 488 nm before and after adding 200 μM Angeli's salt were acquired in 20 mM HEPES buffer (pH 7.4). 996 μL HEPES was added to an Eppendorf tube, then 2 μL of 5 mM XF1, and 2.3 μL of 86 mM Angeli's salt were added. The mixtures were vortexed for 5 seconds. The reaction was monitored every 5 minutes for 30 minutes.

Selectivity. Selectivity for **XF1** was performed by treating it with various reactive species (5 mM glutathione and 200 μM for other species) by monitoring fluorescent change every 5 minutes for 30 minutes with excitation wavelength at 488 nm. Stock solution was prepared as 5 mM in DMSO and the selectivity reactions were performed in 20 mM HEPES (pH 7.4).

9. Inhibition experiments with HCO_3^- and TEMPOL

Different volumes of 100 mM HCO_3^- in DI-H_2O (0 μL, 2 μL, 5 μL, 10 μL and 50 μL) were added to the 20 mM HEPES buffer in the presence of 10 μL of 5 mM isatin (50 μM final concentration) in DMSO, followed by adding 5.29 μL of 37.9 mM $ONOO^-$ (200 μM final concentration). The cuvette was shaken gently to assure mixing. Then, fluorescence spectra of the anthranilic acid product were acquired 1 min after addition with excitation wavelength at 320 nm.

Different volumes of 100 mM TEMPOL in DI-H_2O (0 μL, 2 μL, 5 μL, 10 μL and 50 μL) were added to the 20 mm HEPES buffer in the presence of 10 μL of 5 mM isatin in DMSO (50 μM final

concentration), followed by adding 5.29 µL of 37.9 mM $ONOO^-$ (200 µM final concentration). The cuvette was shaken gently to assure mixing. Then, fluorescence spectra of the anthranilic acid product were acquired 1 min after addition of $ONOO^-$ with excitation wavelength at 320 nm.

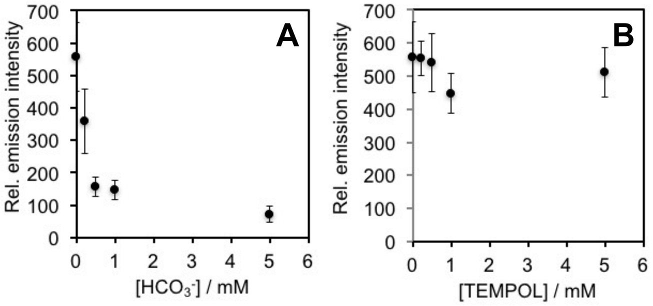

Figure S6. Inhibitor experiments with isatin. (A) Fluorescence emission intensity at 400 nm of 50 µM isatin, 200 µM $ONOO^-$, and 0–5 mM $NaHCO_3$. (B) Fluorescence emission intensity at 400 nm of 50 µM isatin, 200 µM $ONOO^-$, and 0–5 mM TEMPOL. All experiments were performed in 20 mM HEPES (pH 7.4), containing 1% DMSO with λ_{ex} = 320 nm.

10. Inhibition experiments with HCO_3^- and glutathione

Inhibition of response by glutathione

Different volumes of 50 mM glutathione in DI-H_2O (0 µL, 1 µL, 2 µL, 4 µL and 20 µL) were added to the 20 mM HEPES buffer in the presence of 4 µL of 5 mM **PNCL** in DMSO (20 µM final concentration), followed by adding 5.2 µL of 38.4 mM $ONOO^-$ (200 µM final concentration). The cuvette was shaken gently to assure mixing. Then time scans were acquired using the time scan module with emission wavelength at 525 nm.

Different volumes of 50 mM glutathione in DI-H_2O (0 µL, 1 µL, 2 µL, 4 µL and 20 µL) were added to the 20 mM HEPES buffer in the presence of 4 µL of 5 mM **PNCL** in DMSO (20 µM final concentration), followed by adding 1.2 µL of 172.7 mM Angeli's salt (200 µM final concentration). The cuvette was shaken gently to assure mixing. Then time scans were acquired using the time scan module with emission wavelength at 525 nm.

Inhibition of response by HCO_3^-

Different volumes of 50 mM $NaHCO_3$ in DI-H_2O (0 µL, 1 µL, 2 µL, 4 µL and 20 µL) were added to the 20 mM HEPES buffer in the presence of 2 µL of 5 mM **PNCL** in DMSO (20 µM final concentration), followed by adding 5.2 µL of 38.4 mM $ONOO^-$ (200 µM final concentration). The cuvette was shaken gently to assure mixing. Then time scans were acquired using the time scan module with emission wavelength at 525 nm.

Different volumes of 50 mM $NaHCO_3$ in DI-H_2O (0 µL, 20 µL, 40 µL, 60 µL, 80 µL and 100 µL) were added to the 20 mM HEPES buffer in the presence of 4 µL of 5 mM **PNCL** in DMSO (20 µM final concentration), followed by adding 1.1 µL of 177.4 mM Angeli's salt (200 µM final concentration). The cuvette was shaken gently to assure mixing. Then time scans were acquired using the time scan module with emission wavelength at 525 nm.

Inhibition experiments for **XF1**

Different volumes of 50 mM glutathione in DI-H_2O (0 µL, 1 µL, 2 µL, 4 µL and 20 µL) were added to the 20 mM HEPES buffer in the presence of 2 µL of 5 mM **XF1** (10 µM final concentration) in DMSO, followed by adding 1.2 µL of 172.7 mM Angeli's salt. The cuvette was shaken gently to assure mixing. Then time scans were acquired using the time scan module with emission wavelength at 525 nm.

Different volumes of 50 mM $NaHCO_3$ in DI-H_2O (0 µL, 20 µL, 40 µL, 60 µL, 80 µL and 100 µL) were added to the 20 mM HEPES buffer in the presence of 2 µL of 5 mM **XF1** in DMSO (10 µM final concentration), followed by adding 1.1 µL of 177.4 mM Angeli's salt (200 µM final concentration). The cuvette was shaken gently to assure mixing. Then time scans were acquired using the time scan module with emission wavelength at 525 nm.

11. Cell culture

Macrophages (RAW 264.7) were purchased from ATCC and cultured in high glucose Dulbecco's Modified Eagle Media (DMEM) supplemented with 10% Fetal Bovine Serum (FBS) and 1% antibiotics (penicillin/streptomycin, 100 U/mL). Cells were maintained in a humidified incubator at 37 °C with 5% CO_2. 12 hours before the experiment, cells were passed and plated on multi-well plates by adding 1000K–1500K of macrophages per well, filling each well with 1 mL of media.

Cells were maintained in a humidified incubator at 37 °C with 5% CO_2. Two days before the experiment, cells were passed and plated on Costar® 12-well plates by adding 150K–200K of A549 cells per well, filling each well up to 1 mL of media.

Chemiluminescent responses and MTT absorption were measured using a Cytation 5 BioTek plate reader (Winooski, VT). Fluorescent imaging was conducted using an EVOS-fl fluorescent microscope (Advanced Microscopy Group) equipped with a GFP filter cube.

12. MTT assay

3-(4,5-Dimethylthiazol-2-yl)-2,5-diphenyltetrazolium bromide (MTT) assay: RAW 264.7 macrophage cells (10^6 cell/mL) were seeded in a 96-well plate to a total volume of 100 µL/well. The plate was maintained at 37 °C with 5% CO_2 for 12 h. Cells were then incubated for 24 h after adding different concentrations of **PNCL**, 0, 0.1, 1, 10, 100 and 1000 µM respectively. 10 µL of the MTT reagent (Cayman Chemical, Ann Arbor, MI) was then added to each well, and mixed gently. After 4 h incubation, 100 µL of crystal dissolving solution was added to each well to dissolve the formazan crystals. Absorbance was measured at 570 nm in a Cytation 5 BioTek plate reader.[11]

11. W. Huber and J. C. Koella, *Acta Trop*. **1993**, *55*, 257–261.

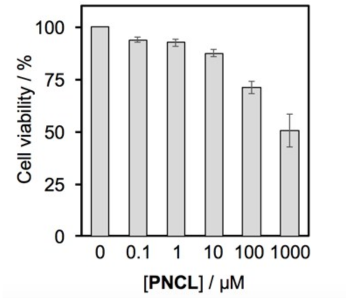

Figure S7. MTT assay of RAW264.7 macrophage cells in the presence of different concentrations of **PNCL**. Error bars are ±S.D. (n = 3).

13. Cellular internalization of PNCL.

RAW264.7 macrophage cells (10^6 cell/mL) were seeded in a 12-well plate to a total volume of 1 mL/well. Plate was maintained at 37 °C with 5% CO_2 for 12 h. Prior to imaging, the medium was removed and cells were washed with 2 x 1 mL PBS, and each well was filled with 992 µL of PBS. Then, 8 µL of 5 mM **PNCL** in DMSO (40 µM final concentration) or 8 µL DMSO was added. The plate was imaged on an EVOS-fl fluorescent microscope using a GFP filter set after 10 min incubation.

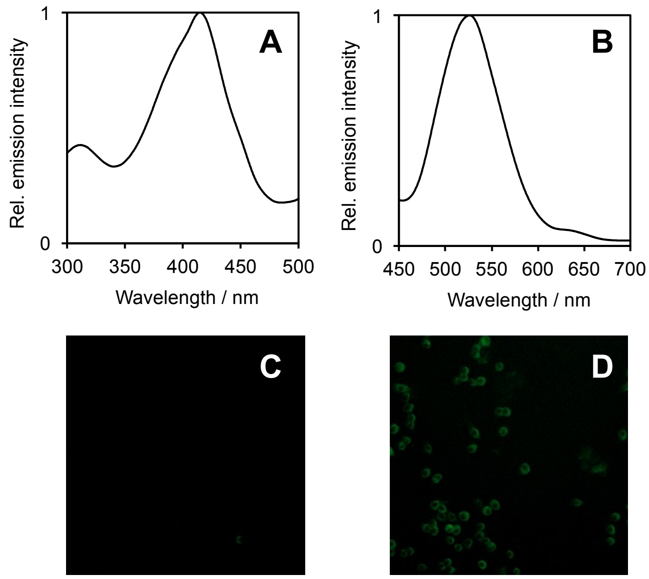

Figure S8. Cellular internalization of **PNCL**. Normalized fluorescence (A) excitation and (B) emission spectrum of **PNCL** in 20 mM HEPES (pH 7.4). Confocal fluorescence images of living RAW 264.7 cells in (C) the absence of **PNCL** or (D) presence of 40 µM **PNCL**.

14. Detection of SIN-1 generated $ONOO^-$ in living cells

<u>Detection of SIN-1 generated $ONOO^-$ in macrophages</u>

RAW 264.7 macrophage cells (10^6 cell/mL) were seeded in a 12-well plate to a total volume of 1 mL/well. Prior to imaging, the medium was removed and cells were washed with 1 x 1 mL PBS.

Each well was filled with 996 µL of FluoroBrite DMEM Media. Then, 4 µL of 5 mM **PNCL** in DMSO (20 µM final concentration) was added to each well, and incubated for 30 min. After incubation, the media was removed and cells were washed with 2 x 1 mL PBS. Different amount of PBS media was added into each well to make the final volume equal to 1 mL. Different volumes of 50 mM SIN-1 in DMSO (0 µL, 8 µL, 16 µL, 20 µL, and 40 µL) were added into each well. Then luminescence response was measure by the Cytation 5 BioTek plate reader by using the luminescence detection method, endpoint read type, and setting gain to 135 and temperature to 37 °C.

<u>Detection of SIN-1 generated ONOO⁻ in A549 cells</u>

Human lung adenocarcinoma epithelial cell (A549) were plated in a 12-well plate to a total volume of 1 mL/well. The media was removed upon 90–95% confluence. Each well was washed with 1 x 1 mL PBS, and each well was filled with 996 µL of FluoroBrite DMEM Media. Then, 4 µL of 5 mM **PNCL** in DMSO (20 µM final concentration) was added to each well, and incubated for 30 min. After incubation, the media was removed and cells were washed with 2 x 1 mL PBS. Different amount of PBS media was added into each well to make the final volume equal to 1 mL. Different volumes of 50 mM SIN-1 in DMSO (0 µL, 8 µL, 16 µL, 20 µL, and 40 µL) were added into each well. Then luminescence response was measure by the plate reader.

<u>Scavenger experiment in macrophages.</u> Macrophages were washed with 1 x 1 mL PBS, and each well was filled with 996 µL of FluoroBrite DMEM Media. Then, 4 µL of 5 mM **PNCL** in DMSO (20 µM final concentration) was added to each well, and incubated for 30. The media was removed after incubation and cells were washed with 2 x 1 mL PBS. Different amount of PBS media was added into each well to make the final volume equal to 1 mL. 40 µL of 50 mM SIN-1 in DMSO, 1.4 µL or 2.8 µL of 2 mg/ mL Mn(III)TMPyP were added.

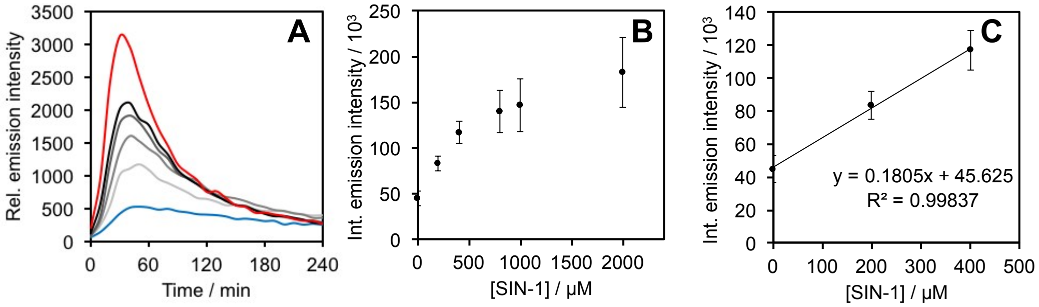

Figure S9. ONOO⁻ detection in A549 cells. (A) Time scans and (B) integrated intensity of chemiluminescence emission of A549 cells incubated with 20 µM **PNCL** for 30 minutes, washed and then treated with 0 (blue trace), 200 µM, 400 µM, 800 µM, 1 mM, and 2 mM (red trace) SIN-1. (C) The linear region of the response curve shown in (B).

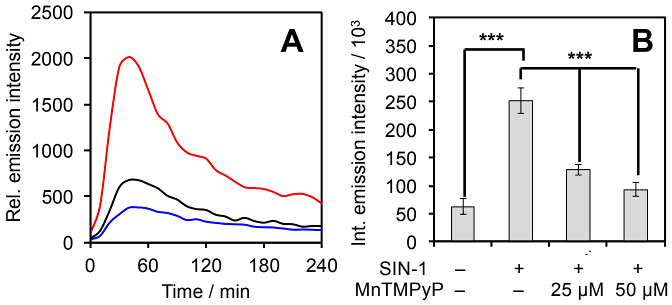

Figure S10. Inhibition of signal from SIN-1 by $ONOO^-$ scavenger MnTMPyP. (A) Time scans of RAW 264.7 macrophages incubated with 20 µM **PNCL** for 30 minutes, washed, and then incubated with (blue trace) vehicle control, (red trace) 2 mM SIN-1, or (black trace) 2 mM SIN-1 and 50 µM Mn(III)TMPyP. (B) Integrated intensity of chemiluminescence emission of RAW 264.7 macrophages incubated with 20 µM **PNCL** for 30 minutes, washed, and then incubated with vehicle control, 2 mM SIN-1, 2 mM SIN-1 and 25 µM MnTMPyP, or 2 mM SIN-1 and 50 µM MnTMPyP. Error bars are S.D. from n = 3 wells. Statistical significance was assessed using a two-tailed student's t-test. *** $p < 0.001$.

15. Application of PNCL for detecting endogenous $ONOO^-$. 0.1 mg/mL LPS was prepared in DI-H_2O, and 2 mg/mL Mn(III)TMPyP solution was made in 20 mM PBS buffer (pH = 7.42) on the same day of the test. Prior to imaging, media was removed from macrophages and macrophages were washed with 1 x 1 mL PBS, and each well was filled with FluoroBrite DMEM Media. 10 µL of LPS (1000 ng/mL final concentration) were added to experimental wells, and the control was performed by treating with vehicle. After 16 h incubation, 4 µL of 5 mM **PNCL** (20 µM final concentration) was added to each well, and incubated for another 30 min. After incubation, the media was removed and cells were washed with 2 x 1 mL PBS, and vehicle or 25 µM Mn(III)TMPyP was added to the appropriate wells. Then the luminescence response was measured every 10 minutes over 4 hours.

16. iNOS inhibition of the production of $ONOO^-$ in macrophages. 0.1 mg/mL LPS and 10 mg/mL 1400W (Cayman Chemical, Ann Arbor, MI) were prepared in DI-H_2O on the same day of the test. Prior to imaging, media was removed from macrophages and cells were washed with 1 x 1 mL PBS, and each well was filled with FluoroBrite DMEM Media. 10 µL of LPS (1000 ng/mL final concentration) and 5 µL of 1400W (200 µM final concentration) were added to the appropriate wells. The control was performed by treating with vehicle. After 16 h incubation, 4 µL of 5 mM **PNCL** (20 µM final concentration) was added to each well, and incubated for another 30 min. The media was removed and cells were washed with 2 x 1 mL PBS. 5 µL of 1400W were added for the inhibition tests. Then the luminescence response was measured every 10 minutes over 4 hours.

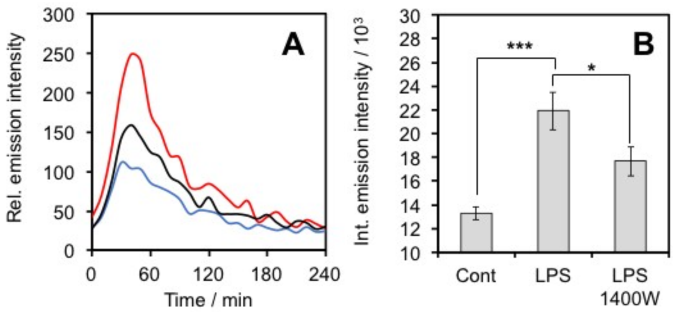

Figure S11. Inhibition of iNOS mediated $ONOO^-$ production. (A) Time scans of RAW 264.7 macrophages incubated with (blue trace) vehicle control, (red trace) 1000 ng/mL LPS, or (black trace) 1000 ng/mL LPS and 200 μM 1400W for 16 hours, then incubated with 20 μM **PNCL** and vehicle control or 1400W for 30 minutes, washed and measured. (B) Integrated intensity of chemiluminescence emission of RAW 264.7 macrophages incubated with vehicle control, 1000 ng/mL LPS, or 1000 ng/mL LPS and 200 μM 1400W for 16 hours, then incubated with 20 μM **PNCL** and vehicle control or 1400W for 30 minutes, washed and measured. Error bars are S.D. from n = 3 wells. Statistical significance was assessed using a two-tailed student's t-test. *** $p < 0.001$, * $p < 0.05$.

17. Scanned ^{1}H and ^{13}C NMR spectra

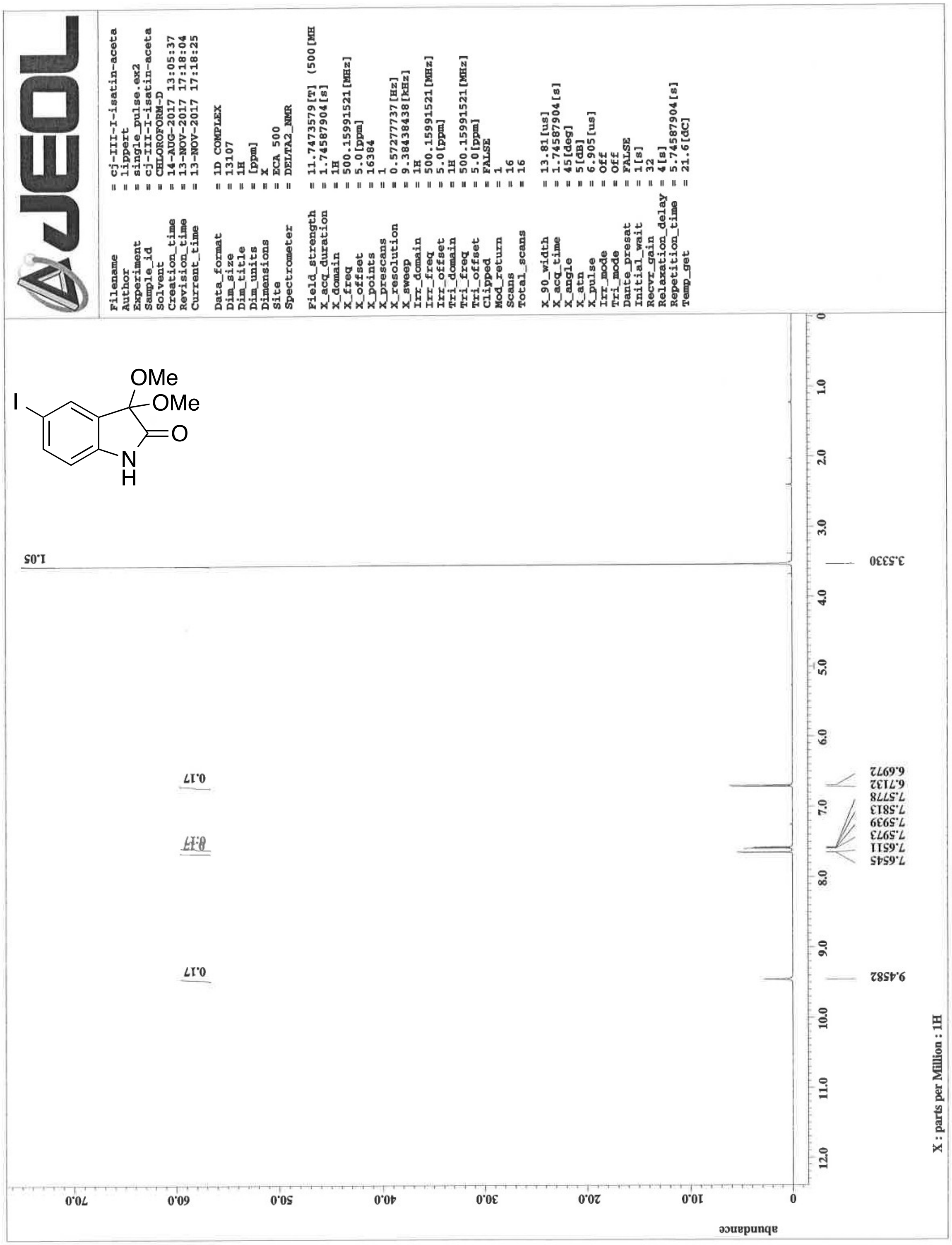

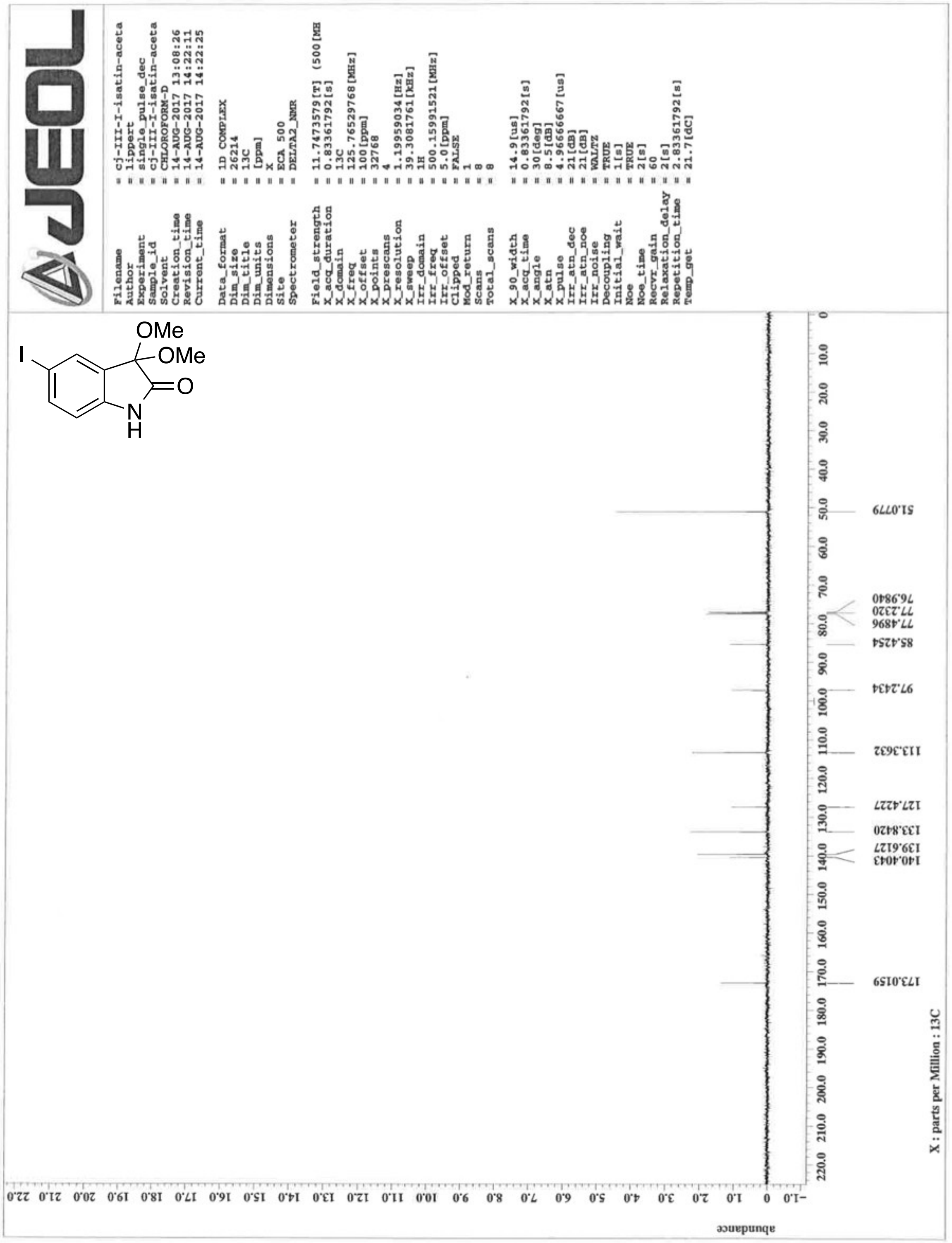
I
OMe
OMe
O
N
H
Filename = cj-III-I-isatin-aceta
Author = lippert
Experiment = single_pulse_dec
Sample_id = cj-III-I-isatin-aceta
Solvent = CHLOROFORM-D
Creation_time = 14-AUG-2017 13:08:26
Revision_time = 14-AUG-2017 14:22:11
Current_time = 14-AUG-2017 14:22:25
Data_format = 1D COMPLEX
Dim_size = 26214
Dim_title = 13C
Dim_units = [ppm]
Dimensions = X
Site = ECA 500
Spectrometer = DELTA2_NMR
Field_strength = 11.7473579[T] (500[MH
X_acq_duration = 0.83361792[s]
X_domain = 13C
X_freq = 125.76529768[MHz]
X_offset = 100[ppm]
X_points = 32768
X_prescans = 4
X_resolution = 1.19959034[Hz]
X_sweep = 39.3081761[kHz]
Irr_domain = 1H
Irr_freq = 500.15991521[MHz]
Irr_offset = 5.0[ppm]
Clipped = FALSE
Mod_return = 1
Scans = 8
Total_scans = 8
X_90_width = 14.9[us]
X_acq_time = 0.83361792[s]
X_angle = 30[deg]
X_atn = 8.5[dB]
X_pulse = 4.96666667[us]
Irr_atn_dec = 21[dB]
Irr_atn_noe = 21[dB]
Irr_noise = WALTZ
Decoupling = TRUE
Initial_wait = 1[s]
Noe = TRUE
Noe_time = 2[s]
Recvr_gain = 60
Relaxation_delay = 2[s]
Repetition_time = 2.83361792[s]
Temp_get = 21.7[dC]
51.0779
76.9840
77.2320
77.4896
85.4254
97.2434
113.3632
127.4227
133.8420
139.6127
140.4043
173.0159
X : parts per Million : 13C
abundance

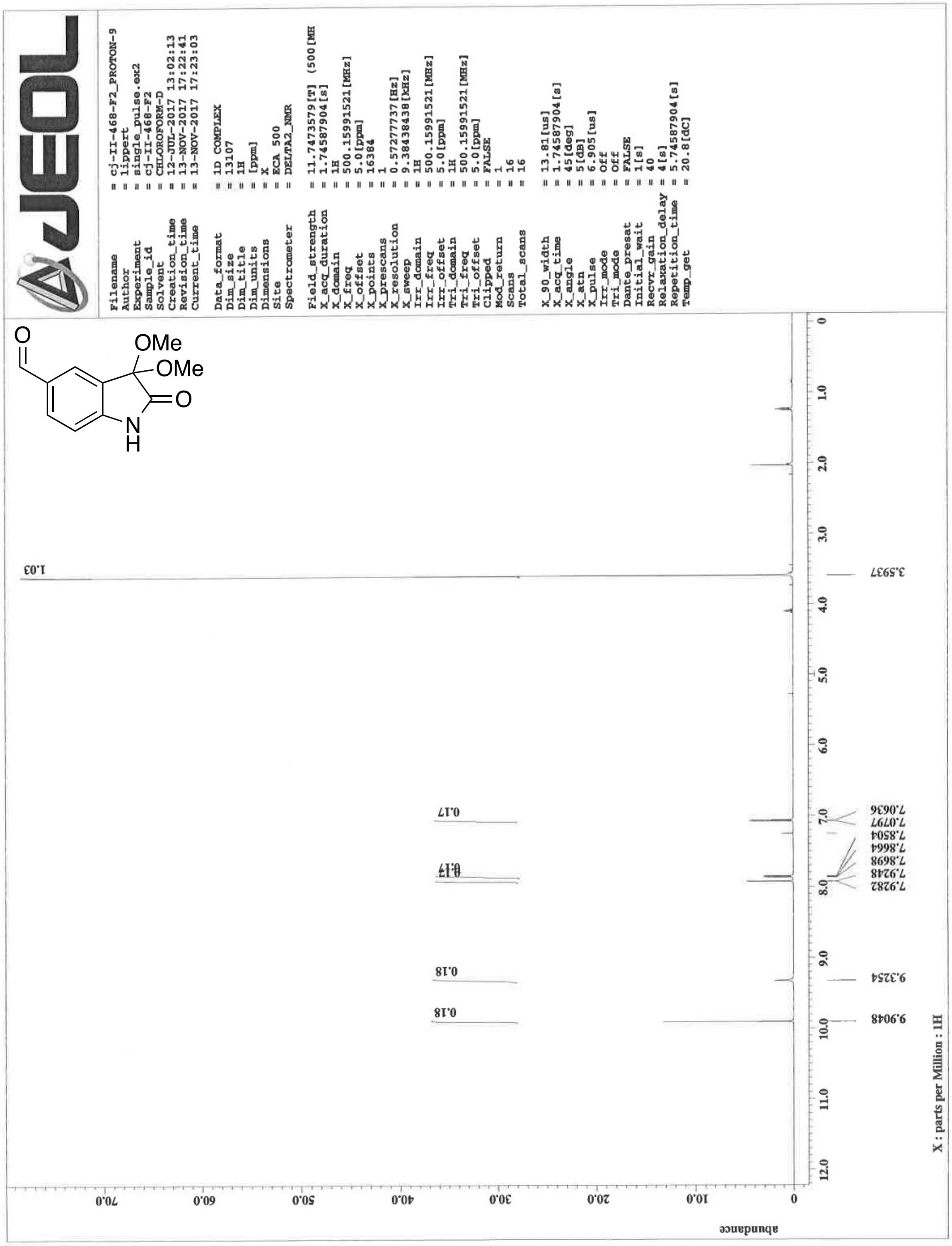
OMe
OMe
O
N
H
Filename = cj-II-468-F2_PROTON-9
Author = lippert
Experiment = single_pulse.ex2
Sample_id = cj-II-468-F2
Solvent = CHLOROFORM-D
Creation_time = 12-JUL-2017 13:02:13
Revision_time = 13-NOV-2017 17:22:41
Current_time = 13-NOV-2017 17:23:03
Data_format = 1D COMPLEX
Dim_size = 13107
Dim_title = 1H
Dim_units = [ppm]
Dimensions = X
Site = ECA 500
Spectrometer = DELTA2_NMR
Field_strength = 11.7473579[T] (500[MH
X_acq_duration = 1.74587904[s]
X_domain = 1H
X_freq = 500.15991521[MHz]
X_offset = 5.0[ppm]
X_points = 16384
X_prescans = 1
X_resolution = 0.57277737[Hz]
X_sweep = 9.38438438[kHz]
Irr_domain = 1H
Irr_freq = 500.15991521[MHz]
Irr_offset = 5.0[ppm]
Tri_domain = 1H
Tri_freq = 500.15991521[MHz]
Tri_offset = 5.0[ppm]
Clipped = FALSE
Mod_return = 1
Scans = 16
Total_scans = 16
X_90_width = 13.81[us]
X_acq_time = 1.74587904[s]
X_angle = 45[deg]
X_atn = 5[dB]
X_pulse = 6.905[us]
Irr_mode = Off
Tri_mode = Off
Dante_presat = FALSE
Initial_wait = 1[s]
Recvr_gain = 40
Relaxation_delay = 4[s]
Repetition_time = 5.74587904[s]
Temp_get = 20.8[dC]
1.03
0.17
0.17
0.18
0.18
3.5937
7.0636
7.0797
7.8504
7.8664
7.8698
7.9248
7.9282
9.3254
9.9048
X : parts per Million : 1H
abundance

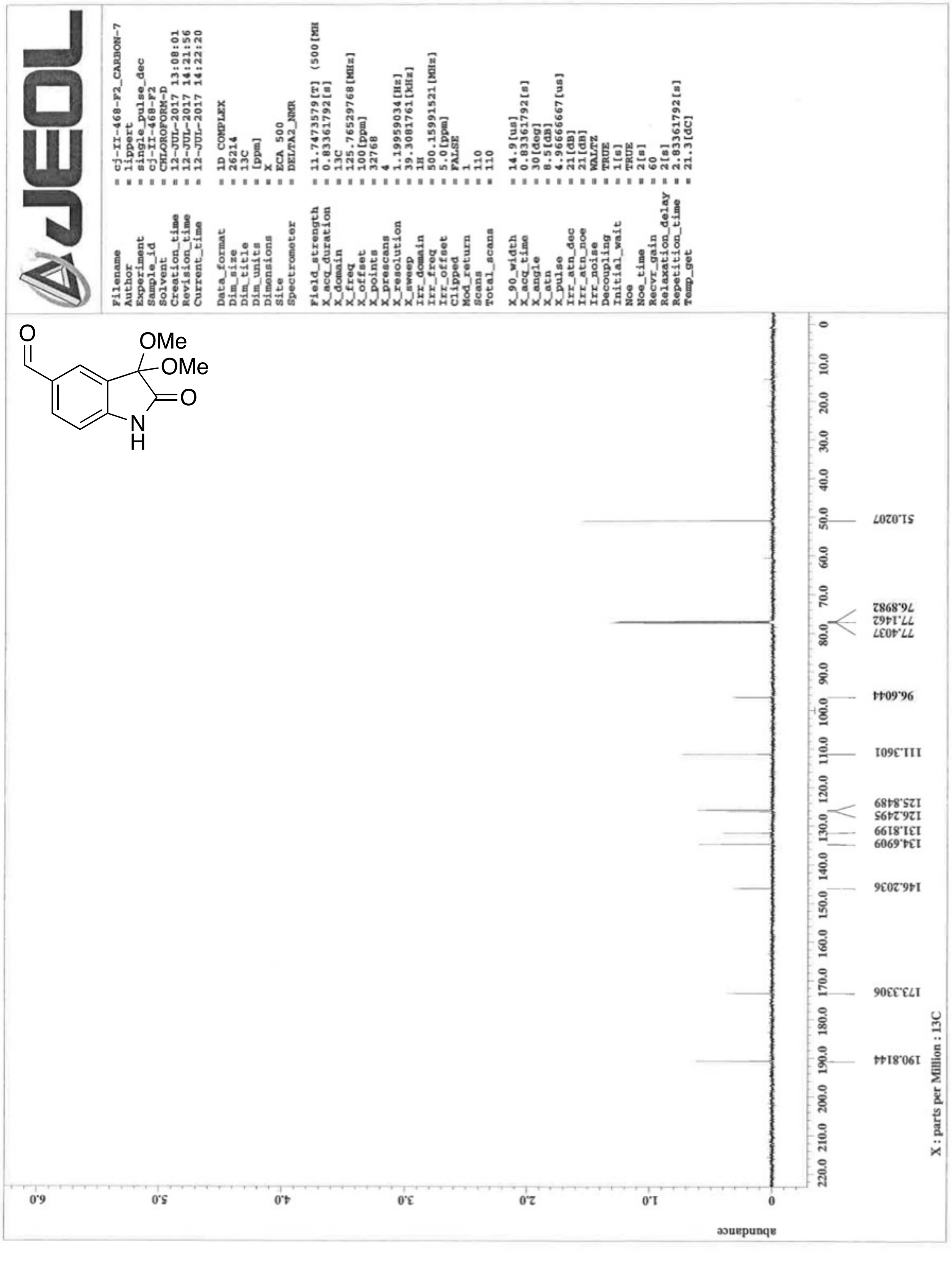

Filename = cj-II-468-F2_CARBON-7
Author = lippert
Experiment = single_pulse_dec
Sample_id = cj-II-468-F2
Solvent = CHLOROFORM-D
Creation_time = 12-JUL-2017 13:08:01
Revision_time = 12-JUL-2017 14:21:56
Current_time = 12-JUL-2017 14:22:20
Data_format = 1D COMPLEX
Dim_size = 26214
Dim_title = 13C
Dim_units = [ppm]
Dimensions = X
Site = ECA 500
Spectrometer = DELTA2_NMR
Field_strength = 11.7473579[T] (500[MH
X_acq_duration = 0.83361792[s]
X_domain = 13C
X_freq = 125.76529768[MHz]
X_offset = 100[ppm]
X_points = 32768
X_prescans = 4
X_resolution = 1.19959034[Hz]
X_sweep = 39.3081761[kHz]
Irr_domain = 1H
Irr_freq = 500.15991521[MHz]
Irr_offset = 5.0[ppm]
Clipped = FALSE
Mod_return = 1
Scans = 110
Total_scans = 110
X_90_width = 14.9[us]
X_acq_time = 0.83361792[s]
X_angle = 30[deg]
X_atn = 8.5[dB]
X_pulse = 4.96666667[us]
Irr_atn_dec = 21[dB]
Irr_atn_noe = 21[dB]
Irr_noise = WALTZ
Decoupling = TRUE
Initial_wait = 1[s]
Noe = TRUE
Noe_time = 2[s]
Recvr_gain = 60
Relaxation_delay = 2[s]
Repetition_time = 2.83361792[s]
Temp_get = 21.3[dC]
OMe
OMe
O
O
N
H
51.0207
76.8982
77.1462
77.4037
96.6044
111.3601
125.8489
126.2495
131.8199
134.6909
146.2036
173.3306
190.8144
X : parts per Million : 13C
abundance

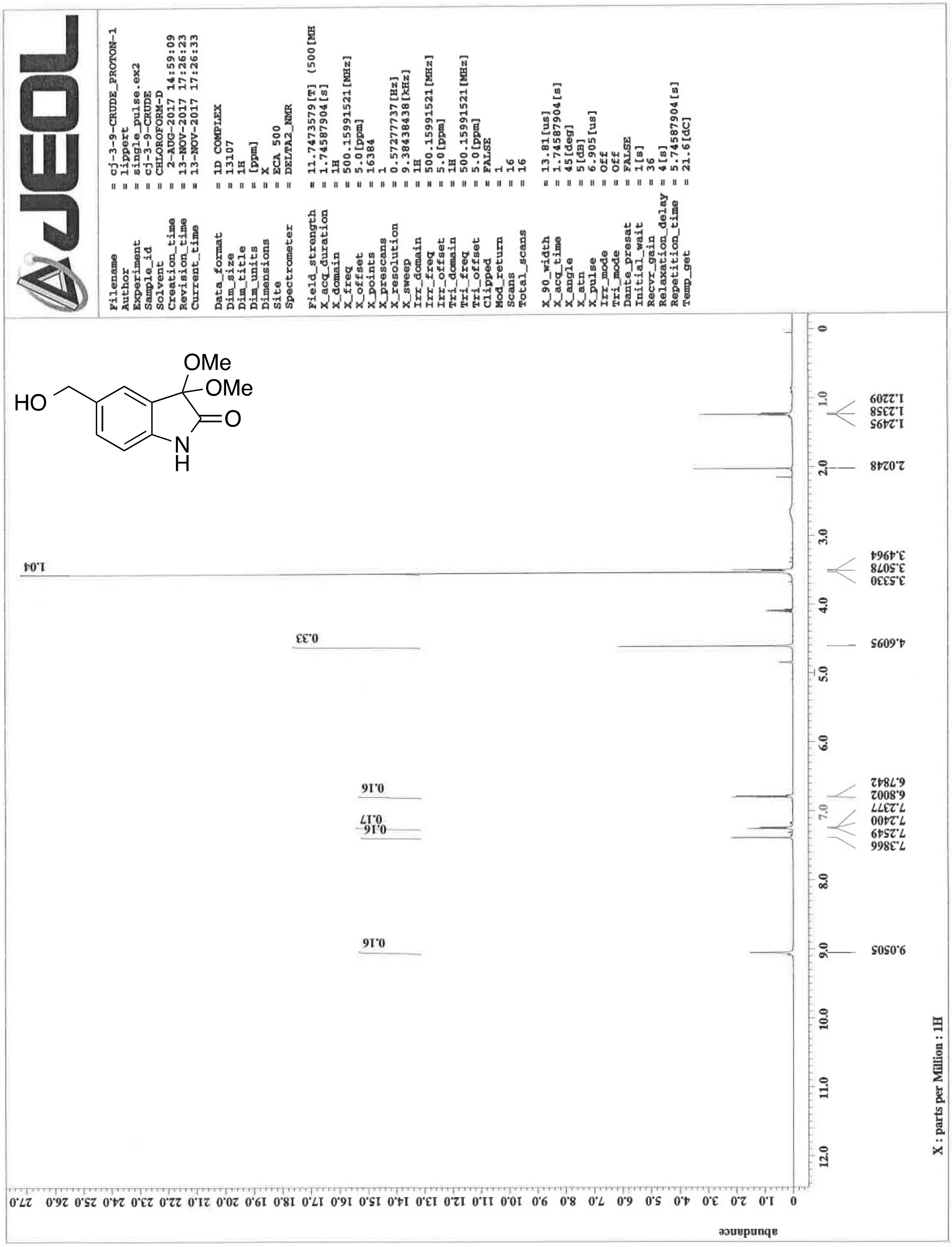
JEOL
Filename = cj-3-9-CRUDE_PROTON-1
Author = lippert
Experiment = single_pulse.ex2
Sample_id = cj-3-9-CRUDE
Solvent = CHLOROFORM-D
Creation_time = 2-AUG-2017 14:59:09
Revision_time = 13-NOV-2017 17:26:23
Current_time = 13-NOV-2017 17:26:33
Data_format = 1D COMPLEX
Dim_size = 13107
Dim_title = 1H
Dim_units = [ppm]
Dimensions = X
Site = ECA 500
Spectrometer = DELTA2_NMR
Field_strength = 11.7473579[T] (500[MH
X_acq_duration = 1.74587904[s]
X_domain = 1H
X_freq = 500.15991521[MHz]
X_offset = 5.0[ppm]
X_points = 16384
X_prescans = 1
X_resolution = 0.57277737[Hz]
X_sweep = 9.38438438[kHz]
Irr_domain = 1H
Irr_freq = 500.15991521[MHz]
Irr_offset = 5.0[ppm]
Tri_domain = 1H
Tri_freq = 500.15991521[MHz]
Tri_offset = 5.0[ppm]
Clipped = FALSE
Mod_return = 1
Scans = 16
Total_scans = 16
X_90_width = 13.81[us]
X_acq_time = 1.74587904[s]
X_angle = 45[deg]
X_atn = 5[dB]
X_pulse = 6.905[us]
Irr_mode = Off
Tri_mode = Off
Dante_presat = FALSE
Initial_wait = 1[s]
Recvr_gain = 36
Relaxation_delay = 4[s]
Repetition_time = 5.74587904[s]
Temp_get = 21.6[dC]
OMe
OMe
HO
O
N
H
1.2209
1.2358
1.2495
2.0248
3.4964
3.5078
3.5330
4.6095
6.7842
6.8002
7.2377
7.2400
7.2549
7.3866
9.0505
1.04
0.33
0.16
0.17
0.16
0.16
X : parts per Million : 1H
abundance

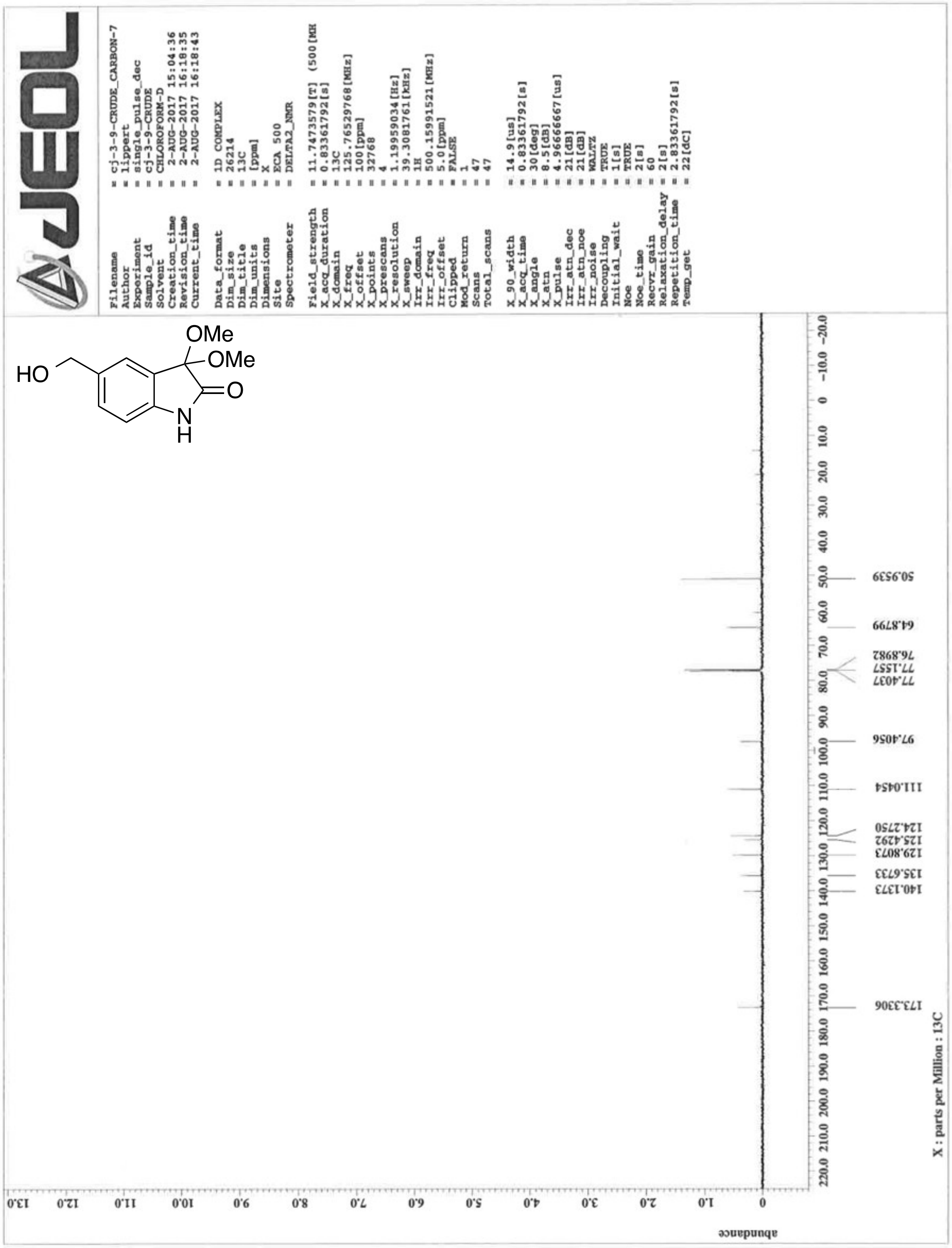

HO
OMe
OMe
O
N
H
JEOL
Filename = cj-3-9-CRUDE_CARBON-7
Author = lippert
Experiment = single_pulse_dec
Sample_id = cj-3-9-CRUDE
Solvent = CHLOROFORM-D
Creation_time = 2-AUG-2017 15:04:36
Revision_time = 2-AUG-2017 16:18:35
Current_time = 2-AUG-2017 16:18:43
Data_format = 1D COMPLEX
Dim_size = 26214
Dim_title = 13C
Dim_units = [ppm]
Dimensions = X
Site = ECA 500
Spectrometer = DELTA2_NMR
Field_strength = 11.7473579[T] (500[MH
X_acq_duration = 0.83361792[s]
X_domain = 13C
X_freq = 125.76529768[MHz]
X_offset = 100[ppm]
X_points = 32768
X_prescans = 4
X_resolution = 1.19959034[Hz]
X_sweep = 39.3081761[kHz]
Irr_domain = 1H
Irr_freq = 500.15991521[MHz]
Irr_offset = 5.0[ppm]
Clipped = FALSE
Mod_return = 1
Scans = 47
Total_scans = 47
X_90_width = 14.9[us]
X_acq_time = 0.83361792[s]
X_angle = 30[deg]
X_atn = 8.5[dB]
X_pulse = 4.96666667[us]
Irr_atn_dec = 21[dB]
Irr_atn_noe = 21[dB]
Irr_noise = WALTZ
Decoupling = TRUE
Initial_wait = 1[s]
Noe = TRUE
Noe_time = 2[s]
Recvr_gain = 60
Relaxation_delay = 2[s]
Repetition_time = 2.83361792[s]
Temp_get = 22[dC]
50.9539
64.8799
76.8982
77.1557
77.4037
97.4056
111.0454
124.2750
125.4292
129.8073
135.6733
140.1373
173.3306
X : parts per Million : 13C
abundance

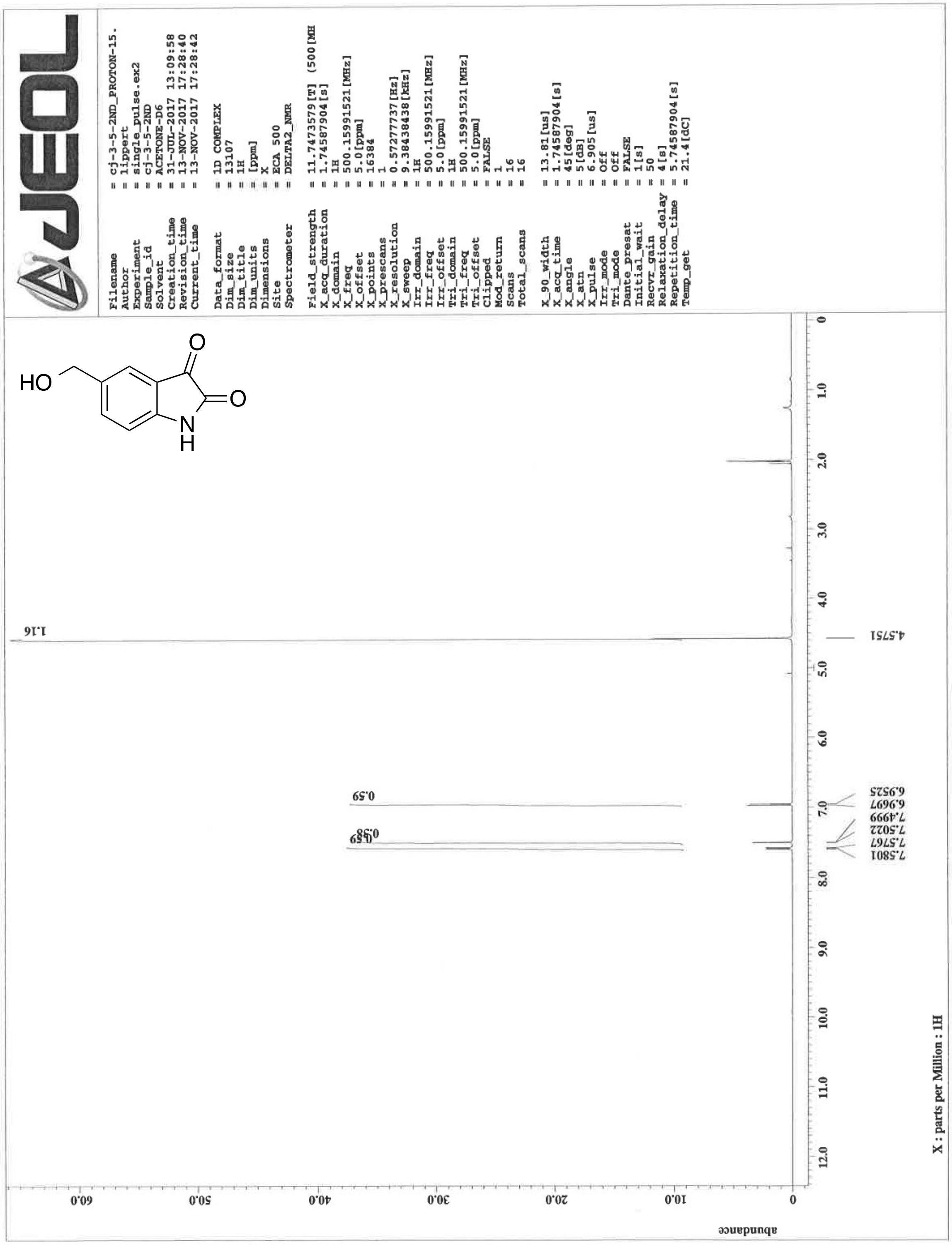

Filename = cj-3-5-2ND_PROTON-15.
Author = lippert
Experiment = single_pulse.ex2
Sample_id = cj-3-5-2ND
Solvent = ACETONE-D6
Creation_time = 31-JUL-2017 13:09:58
Revision_time = 13-NOV-2017 17:28:40
Current_time = 13-NOV-2017 17:28:42
Data_format = 1D COMPLEX
Dim_size = 13107
Dim_title = 1H
Dim_units = [ppm]
Dimensions = X
Site = ECA 500
Spectrometer = DELTA2_NMR
Field_strength = 11.7473579[T] (500[MH
X_acq_duration = 1.74587904[s]
X_domain = 1H
X_freq = 500.15991521[MHz]
X_offset = 5.0[ppm]
X_points = 16384
X_prescans = 1
X_resolution = 0.57277737[Hz]
X_sweep = 9.38438438[kHz]
Irr_domain = 1H
Irr_freq = 500.15991521[MHz]
Irr_offset = 5.0[ppm]
Tri_domain = 1H
Tri_freq = 500.15991521[MHz]
Tri_offset = 5.0[ppm]
Clipped = FALSE
Mod_return = 1
Scans = 16
Total_scans = 16
X_90_width = 13.81[us]
X_acq_time = 1.74587904[s]
X_angle = 45[deg]
X_atn = 5[dB]
X_pulse = 6.905[us]
Irr_mode = Off
Tri_mode = Off
Dante_presat = FALSE
Initial_wait = 1[s]
Recvr_gain = 50
Relaxation_delay = 4[s]
Repetition_time = 5.74587904[s]
Temp_get = 21.4[dC]
HO
O
O
N
H
1.16
0.59
0.58
0.59
4.5751
6.9525
6.9697
7.4999
7.5022
7.5767
7.5801
X : parts per Million : 1H
abundance

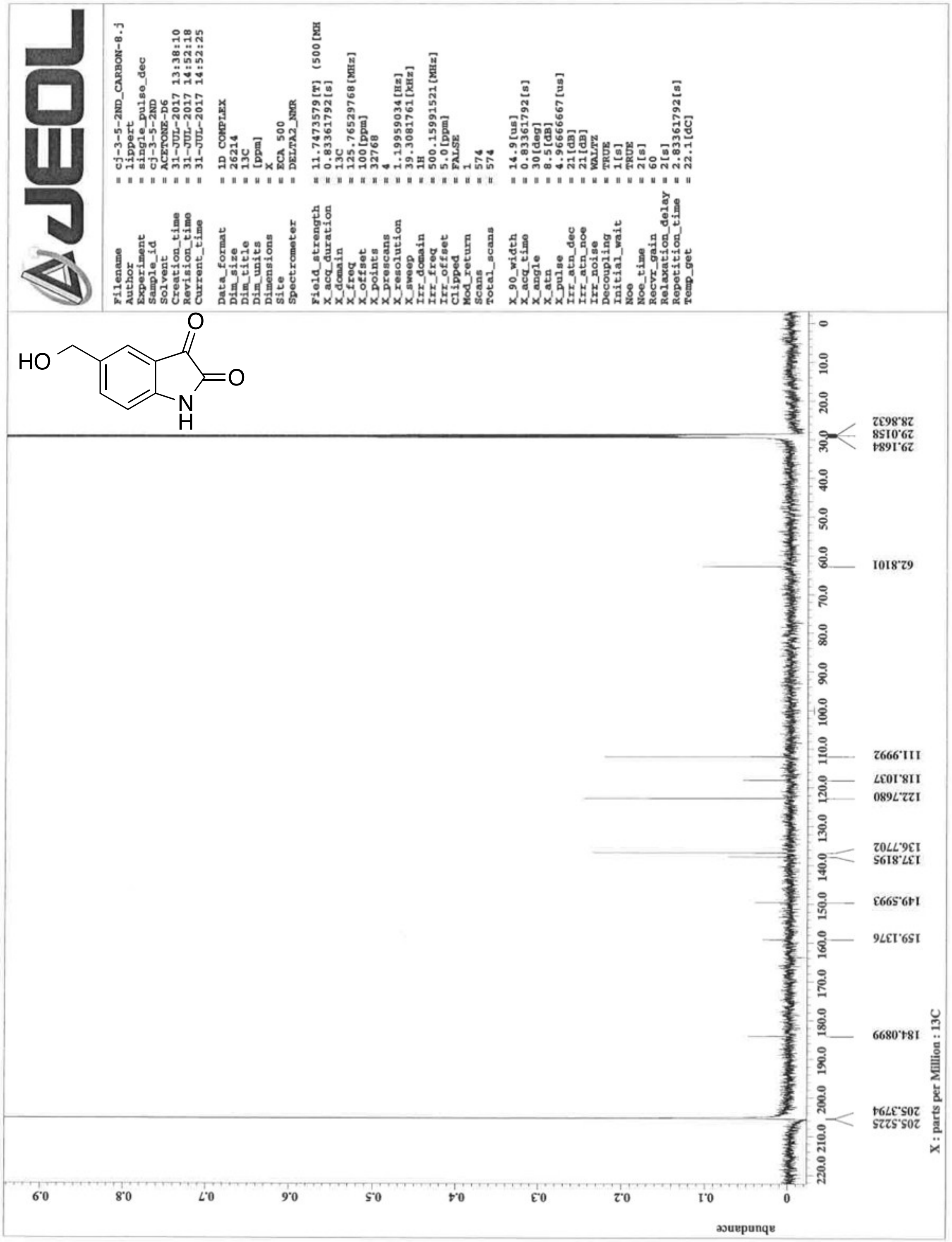
JEOL
Filename = cj-3-5-2ND_CARBON-8.jdf
Author = lippert
Experiment = single_pulse_dec
Sample_id = cj-3-5-2ND
Solvent = ACETONE-D6
Creation_time = 31-JUL-2017 13:38:10
Revision_time = 31-JUL-2017 14:52:18
Current_time = 31-JUL-2017 14:52:25
Data_format = 1D COMPLEX
Dim_size = 26214
Dim_title = 13C
Dim_units = [ppm]
Dimensions = X
Site = ECA 500
Spectrometer = DELTA2_NMR
Field_strength = 11.7473579[T] (500[MH
X_acq_duration = 0.83361792[s]
X_domain = 13C
X_freq = 125.76529768[MHz]
X_offset = 100[ppm]
X_points = 32768
X_prescans = 4
X_resolution = 1.19959034[Hz]
X_sweep = 39.3081761[kHz]
Irr_domain = 1H
Irr_freq = 500.15991521[MHz]
Irr_offset = 5.0[ppm]
Clipped = FALSE
Mod_return = 1
Scans = 574
Total_scans = 574
X_90_width = 14.9[us]
X_acq_time = 0.83361792[s]
X_angle = 30[deg]
X_atn = 8.5[dB]
X_pulse = 4.96666667[us]
Irr_atn_dec = 21[dB]
Irr_atn_noe = 21[dB]
Irr_noise = WALTZ
Decoupling = TRUE
Initial_wait = 1[s]
Noe = TRUE
Noe_time = 2[s]
Recvr_gain = 60
Relaxation_delay = 2[s]
Repetition_time = 2.83361792[s]
Temp_get = 22.1[dC]
HO
O
O
N
H
28.8632
29.0158
29.1684
62.8101
111.9992
118.1037
122.7680
136.7702
137.8195
149.5993
159.1376
184.0899
205.3794
205.5225
X : parts per Million : 13C
abundance

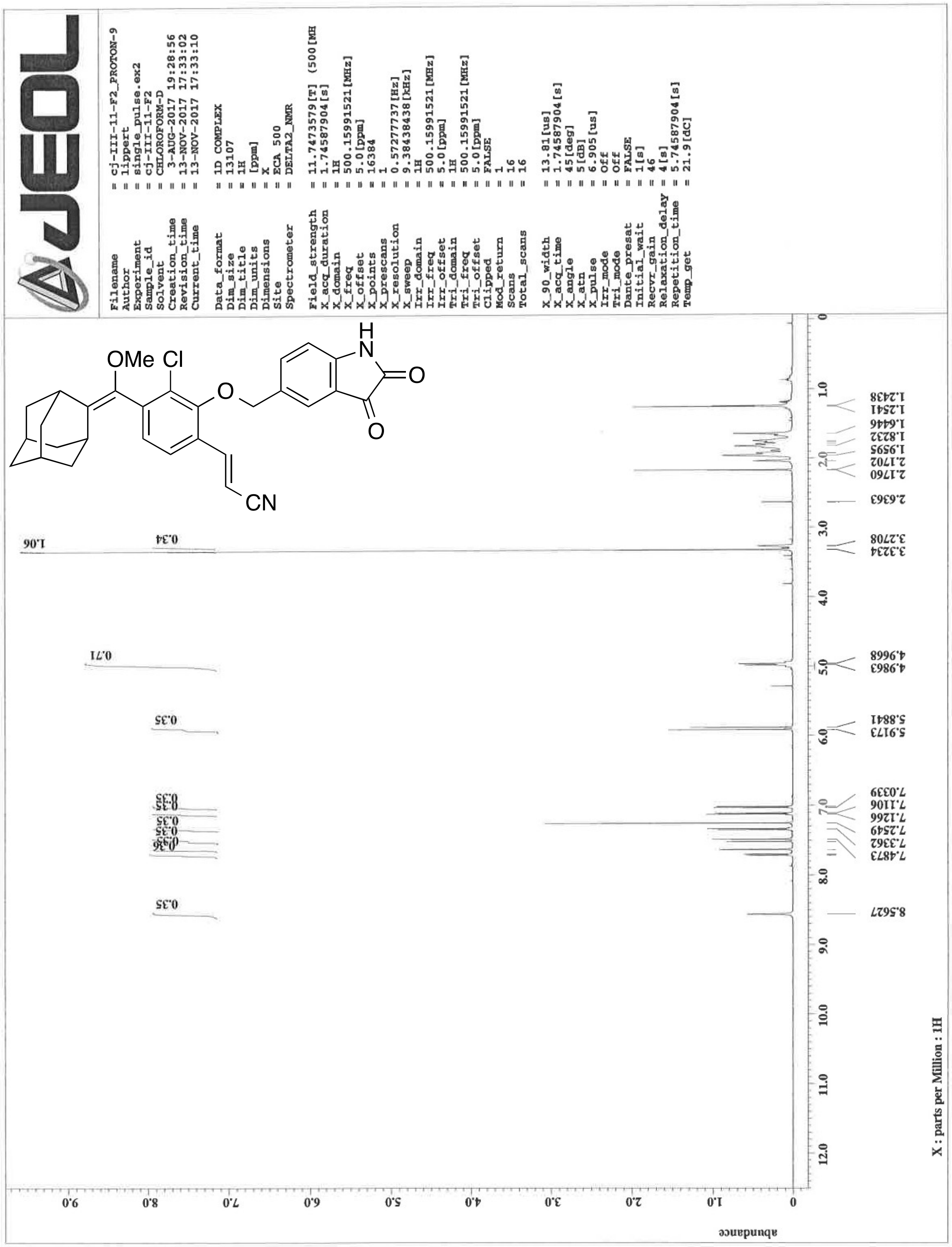
JEOL
Filename = cj-III-11-F2_PROTON-9
Author = lippert
Experiment = single_pulse.ex2
Sample_id = cj-III-11-F2
Solvent = CHLOROFORM-D
Creation_time = 3-AUG-2017 19:28:56
Revision_time = 13-NOV-2017 17:33:02
Current_time = 13-NOV-2017 17:33:10
Data_format = 1D COMPLEX
Dim_size = 13107
Dim_title = 1H
Dim_units = [ppm]
Dimensions = X
Site = ECA 500
Spectrometer = DELTA2_NMR
Field_strength = 11.7473579[T] (500[MH
X_acq_duration = 1.74587904[s]
X_domain = 1H
X_freq = 500.15991521[MHz]
X_offset = 5.0[ppm]
X_points = 16384
X_prescans = 1
X_resolution = 0.57277737[Hz]
X_sweep = 9.38438438[kHz]
Irr_domain = 1H
Irr_freq = 500.15991521[MHz]
Irr_offset = 5.0[ppm]
Tri_domain = 1H
Tri_freq = 500.15991521[MHz]
Tri_offset = 5.0[ppm]
Clipped = FALSE
Mod_return = 1
Scans = 16
Total_scans = 16
X_90_width = 13.81[us]
X_acq_time = 1.74587904[s]
X_angle = 45[deg]
X_atn = 5[dB]
X_pulse = 6.905[us]
Irr_mode = Off
Tri_mode = Off
Dante_presat = FALSE
Initial_wait = 1[s]
Recvr_gain = 46
Relaxation_delay = 4[s]
Repetition_time = 5.74587904[s]
Temp_get = 21.9[dC]
OMe
Cl
O
H
N
O
O
CN
1.2438
1.2541
1.6446
1.8232
1.9595
2.1702
2.1760
2.6363
3.2708
3.3234
4.9668
4.9863
5.8841
5.9173
7.0339
7.1106
7.1266
7.2549
7.3362
7.4873
8.5627
X : parts per Million : 1H
abundance

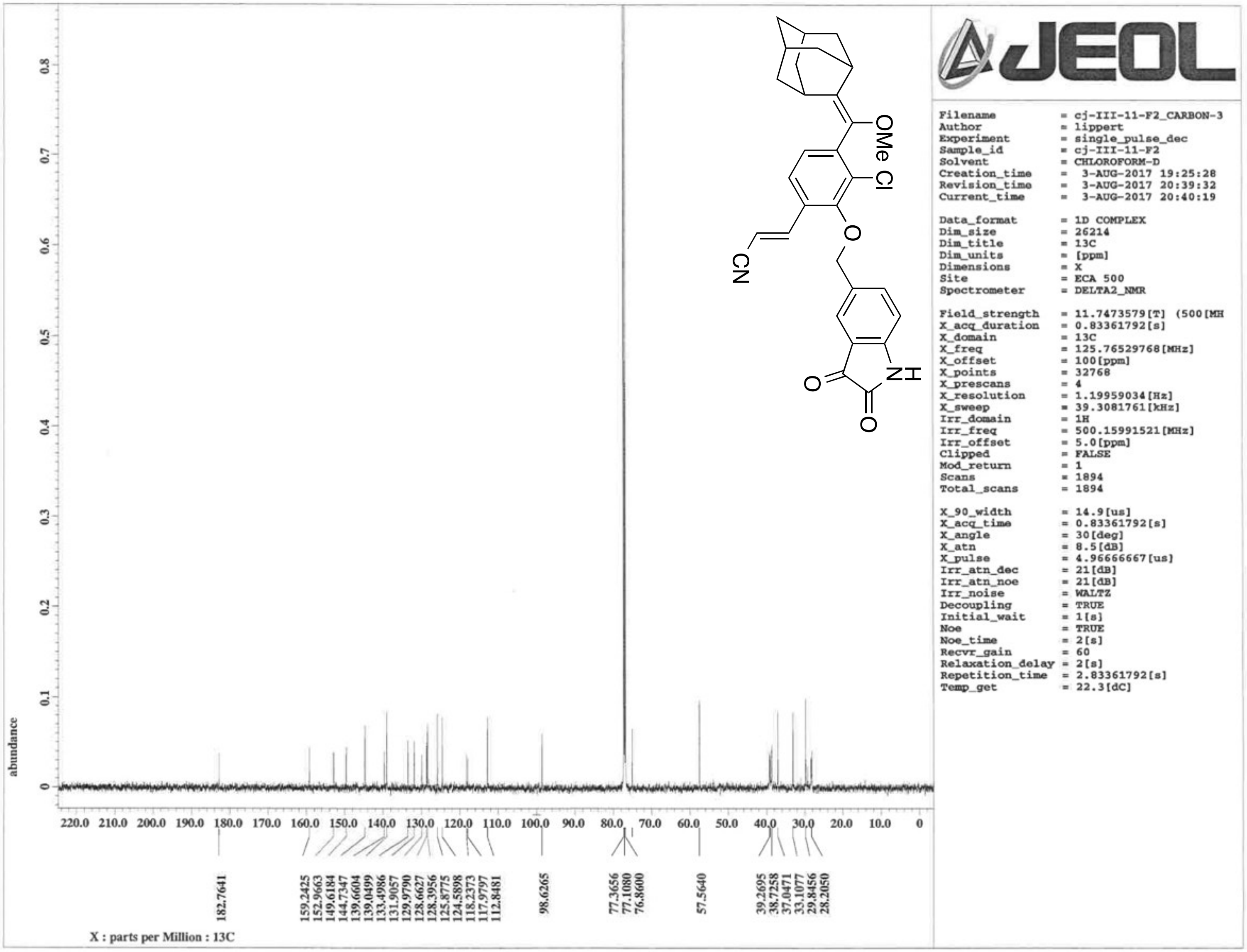
JEOL
Filename = cj-III-11-F2_CARBON-3
Author = lippert
Experiment = single_pulse_dec
Sample_id = cj-III-11-F2
Solvent = CHLOROFORM-D
Creation_time = 3-AUG-2017 19:25:28
Revision_time = 3-AUG-2017 20:39:32
Current_time = 3-AUG-2017 20:40:19
Data_format = 1D COMPLEX
Dim_size = 26214
Dim_title = 13C
Dim_units = [ppm]
Dimensions = X
Site = ECA 500
Spectrometer = DELTA2_NMR
Field_strength = 11.7473579[T] (500[MH
X_acq_duration = 0.83361792[s]
X_domain = 13C
X_freq = 125.76529768[MHz]
X_offset = 100[ppm]
X_points = 32768
X_prescans = 4
X_resolution = 1.19959034[Hz]
X_sweep = 39.3081761[kHz]
Irr_domain = 1H
Irr_freq = 500.15991521[MHz]
Irr_offset = 5.0[ppm]
Clipped = FALSE
Mod_return = 1
Scans = 1894
Total_scans = 1894
X_90_width = 14.9[us]
X_acq_time = 0.83361792[s]
X_angle = 30[deg]
X_atn = 8.5[dB]
X_pulse = 4.96666667[us]
Irr_atn_dec = 21[dB]
Irr_atn_noe = 21[dB]
Irr_noise = WALTZ
Decoupling = TRUE
Initial_wait = 1[s]
Noe = TRUE
Noe_time = 2[s]
Recvr_gain = 60
Relaxation_delay = 2[s]
Repetition_time = 2.83361792[s]
Temp_get = 22.3[dC]
OMe
Cl
O
CN
N
H
O
O
abundance
X : parts per Million : 13C
182.7641
159.2425
152.9663
149.6184
144.7347
139.6604
139.0499
133.4986
131.9057
129.9790
128.6627
128.3956
125.8775
124.5898
118.2373
117.9797
112.8481
98.6265
77.3656
77.1080
76.8600
57.5640
39.2695
38.7258
37.0471
33.1077
29.8456
28.2050

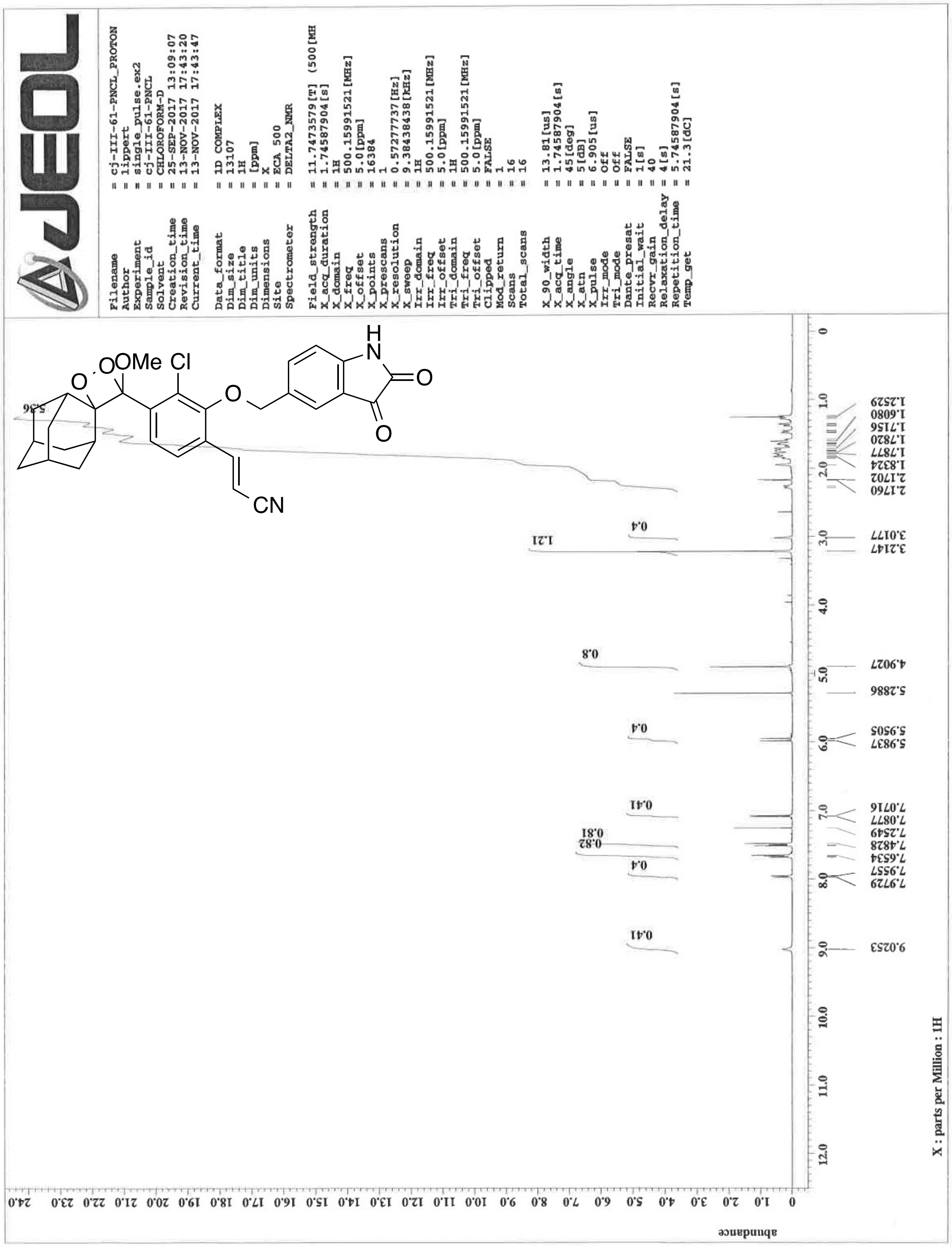
JEOL
Filename = cj-III-61-PNCL_PROTON
Author = lippert
Experiment = single_pulse.ex2
Sample_id = cj-III-61-PNCL
Solvent = CHLOROFORM-D
Creation_time = 25-SEP-2017 13:09:07
Revision_time = 13-NOV-2017 17:43:20
Current_time = 13-NOV-2017 17:43:47
Data_format = 1D COMPLEX
Dim_size = 13107
Dim_title = 1H
Dim_units = [ppm]
Dimensions = X
Site = ECA 500
Spectrometer = DELTA2_NMR
Field_strength = 11.7473579[T] (500[MH
X_acq_duration = 1.74587904[s]
X_domain = 1H
X_freq = 500.15991521[MHz]
X_offset = 5.0[ppm]
X_points = 16384
X_prescans = 1
X_resolution = 0.57277737[Hz]
X_sweep = 9.38438438[kHz]
Irr_domain = 1H
Irr_freq = 500.15991521[MHz]
Irr_offset = 5.0[ppm]
Tri_domain = 1H
Tri_freq = 500.15991521[MHz]
Tri_offset = 5.0[ppm]
Clipped = FALSE
Mod_return = 1
Scans = 16
Total_scans = 16
X_90_width = 13.81[us]
X_acq_time = 1.74587904[s]
X_angle = 45[deg]
X_atn = 5[dB]
X_pulse = 6.905[us]
Irr_mode = Off
Tri_mode = Off
Dante_presat = FALSE
Initial_wait = 1[s]
Recvr_gain = 40
Relaxation_delay = 4[s]
Repetition_time = 5.74587904[s]
Temp_get = 21.3[dC]
OMe
Cl
O
H
N
CN
abundance
X : parts per Million : 1H
0.41
0.4
0.82
0.81
0.41
0.4
0.8
1.21
0.4
5.36
9.0253
7.9729
7.9557
7.6534
7.4828
7.2549
7.0877
7.0716
5.9837
5.9505
5.2886
4.9027
3.2147
3.0177
2.1760
2.1702
1.8324
1.7877
1.7820
1.7156
1.6080
1.2529

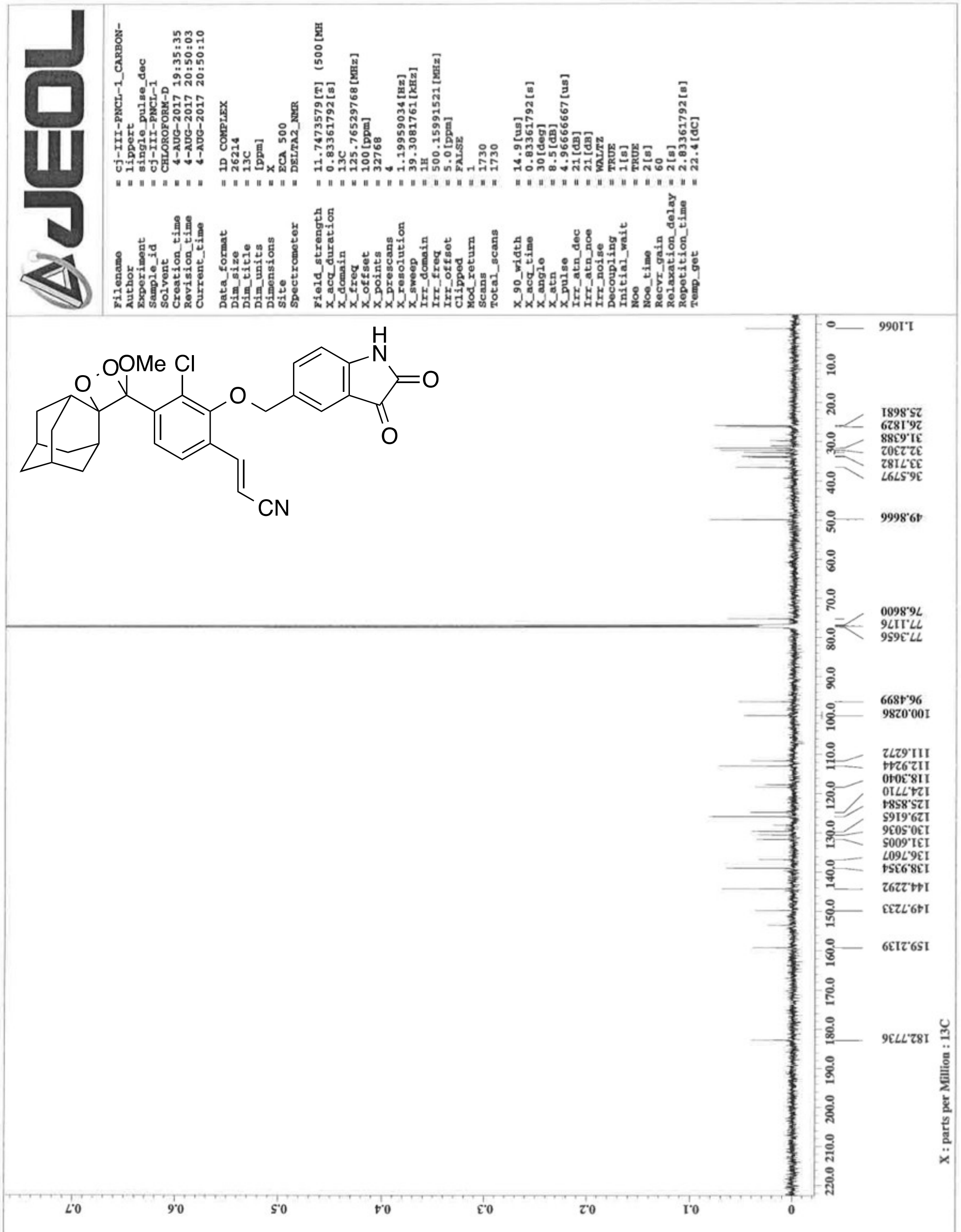

JEOL
Filename = cj-III-PNCL-1_CARBON-
Author = lippert
Experiment = single_pulse_dec
Sample_id = cj-III-PNCL-1
Solvent = CHLOROFORM-D
Creation_time = 4-AUG-2017 19:35:35
Revision_time = 4-AUG-2017 20:50:03
Current_time = 4-AUG-2017 20:50:10
Data_format = 1D COMPLEX
Dim_size = 26214
Dim_title = 13C
Dim_units = [ppm]
Dimensions = X
Site = ECA 500
Spectrometer = DELTA2_NMR
Field_strength = 11.7473579[T] (500[MH
X_acq_duration = 0.83361792[s]
X_domain = 13C
X_freq = 125.76529768[MHz]
X_offset = 100[ppm]
X_points = 32768
X_prescans = 4
X_resolution = 1.19959034[Hz]
X_sweep = 39.3081761[kHz]
Irr_domain = 1H
Irr_freq = 500.15991521[MHz]
Irr_offset = 5.0[ppm]
Clipped = FALSE
Mod_return = 1
Scans = 1730
Total_scans = 1730
X_90_width = 14.9[us]
X_acq_time = 0.83361792[s]
X_angle = 30[deg]
X_atn = 8.5[dB]
X_pulse = 4.96666667[us]
Irr_atn_dec = 21[dB]
Irr_atn_noe = 21[dB]
Irr_noise = WALTZ
Decoupling = TRUE
Initial_wait = 1[s]
Noe = TRUE
Noe_time = 2[s]
Recvr_gain = 60
Relaxation_delay = 2[s]
Repetition_time = 2.83361792[s]
Temp_get = 22.4[dC]
OMe
Cl
O
O
O
H
N
O
O
CN
1.1066
25.8681
26.1829
31.6388
32.2302
33.7182
36.5797
49.8666
76.8600
77.1176
77.3656
96.4899
100.0286
111.6272
112.9244
118.3040
124.7710
125.8584
129.6165
130.5036
131.6005
136.7607
138.9354
144.2292
149.7233
159.2139
182.7736
X : parts per Million : 13C
abundance
